# Multiplexed Nanoscopy via Buffer Exchange

**DOI:** 10.1021/acsnano.4c06829

**Published:** 2024-08-15

**Authors:** Ting-Jui Ben Chang, T. Tony Yang

**Affiliations:** † Department of Electrical Engineering, National Taiwan University, Taipei 10617, Taiwan; ‡ Graduate Institute of Biomedical Electronics and Bioinformatics, National Taiwan University, Taipei 10617, Taiwan; § Department of Physics, National Taiwan University, Taipei 10617, Taiwan; ∥ Nano Science and Technology Program, Taiwan International Graduate Program, Academia Sinica and National Taiwan University, Taipei 10617, Taiwan

**Keywords:** superresolution microscopy, SMLM, (d)STORM, multicolor, multiplexed imaging, expansion microscopy (ExM)

## Abstract

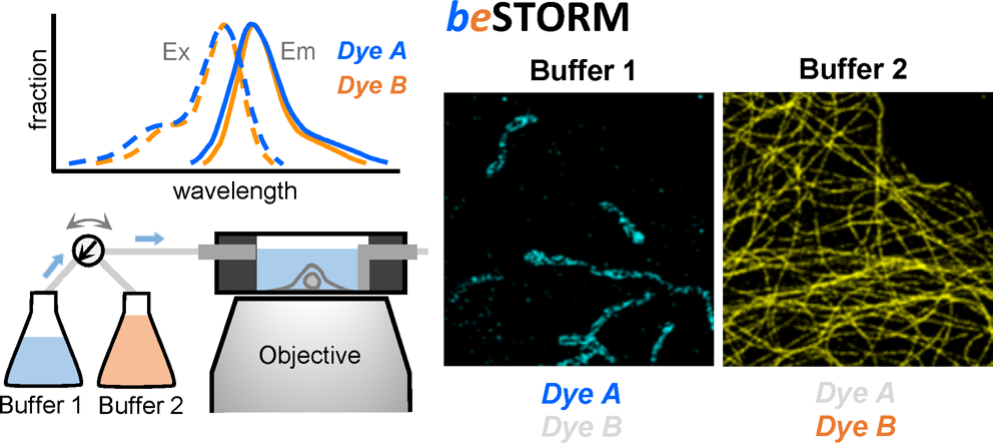

Understanding cellular functions, particularly in their intricate complexity, can greatly benefit from the spatial mapping of diverse molecules through multitarget single-molecule localization microscopy (SMLM). Existing methodologies, primarily restricting the encoding dimensions to color and lifetime or requiring cyclic staining, often involve broad chromatic detection, specialized optical configurations, or sophisticated labeling techniques. Here, we propose a simple approach called buffer-exchange stochastic optical reconstruction microscopy (beSTORM), which introduces an additional dimension to differentiate between single molecules irrespective of their spectral properties. This method leverages the distinguishable photoblinking responses to distinct buffer conditions, offering a straightforward yet effective means of fluorophore discrimination. Through buffer exchanges, beSTORM achieves multitarget SMLM imaging with minimal crosstalk. Direct integration with expansion microscopy (ExM) demonstrates its capability to resolve up to six proteins at the molecular level within a single emission color without chromatic aberration. Overall, beSTORM presents a highly compatible imaging platform, promising significant advancements in highly multiplexed nanoscopy for exploring multiple targets in biological systems with nanoscale precision.

Single-molecule localization microscopy (SMLM), characterized by nanoscale optical spatial resolution,^1^ such as (f)PALM,^2,3^ (d)STORM,^4,5^ and (DNA-)PAINT,^6,7^ serves as a potent tool for exploring organelles and subcellular structures in diverse biological research.^8^ Broadening its capabilities to image multiple targets becomes essential for unraveling spatial relationships among various molecules with diffraction-unlimited details.^9,10^ A common methodology to implement multitarget SMLM involves labeling several molecules in different colors. One direct approach included sequential imaging using distinct dyes with broad spectral separation (∼90 nm peak separation) across the range of visible wavelengths.^11,12^ However, this method could potentially introduce noticeable chromatic aberration under nanoscopic scrutiny.^13^ An alternative method proposed splitting single-molecule fluorescence into several optical paths to facilitate color identification of dyes with partially overlapping spectra (∼20 nm peak separation) using ratiometric detection.^14−16^ A derivative configuration prioritized the excitation spectrum, using three illumination lasers to distinguish four spectrally close far-red fluorophores.^17^ One other category directly resolved the dispersed spectra of fluorophores with either a prism or a grating to distinguish four far-red dyes.^18,19^

Beyond those spectrum-based approaches, the laser-scanning fluorescence-lifetime SMLM provided a two-target imaging solution by differentiating two labels on their lifetime dimension,^20,21^ irrespective of their spectral properties. Other possible solutions were based on the serial labeling, which involved iterative immunostaining steps^22−25^ or DNA-PAINT setup,^26−29^ enabling multitarget SMLM using identical fluorophores. However, these could extend experimental durations or necessitate sophisticated experimental designs. Collectively, while various multitarget SMLM strategies exist leveraging distinct dimensions, they often come with specific demands like complex optical setups, signal unmixing, cyclic sample staining, intricate experimental designs, or division of camera detection fields, thereby presenting implementation hurdles. Moreover, some methods inherently pose challenges when attempting to integrate them with other techniques.

In this study, we introduce a concept to distinguish between single fluorophores emitting at the identical spectrum, thereby contributing an additional dimension to multitarget STORM. Our method is implemented through simple buffer exchanges (termed beSTORM), requiring no further modification of the optical system or additional image processing for fluorophore identification. By capitalizing on the responsive blinking behaviors of fluorophores influenced by buffer surroundings, we achieved multitarget beSTORM imaging using a single laser. Furthermore, by multiplexing with different spectral regimes, we proposed a streamlined four-target dSTORM imaging solution with low crosstalk, necessitating only a buffer-exchange chamber. It exhibits promise for seamless integration into any SMLM imaging optical system. Next, we combined expansion microscopy (ExM)^30,31^ and colabeling strategy with beSTORM to extend the coverage of detectable targets via molecular-level protein characterization in individual buffer channels. This allowed the differentiation of up to six proteins within a single emission color, devoid of chromatic aberration. Significantly, given the additional dimension through simple implementation, beSTORM holds the potential for a wide variety of highly multiplexed SMLM imaging.

## Results and Discussion

### Principle of beSTORM

**Figure 1:**
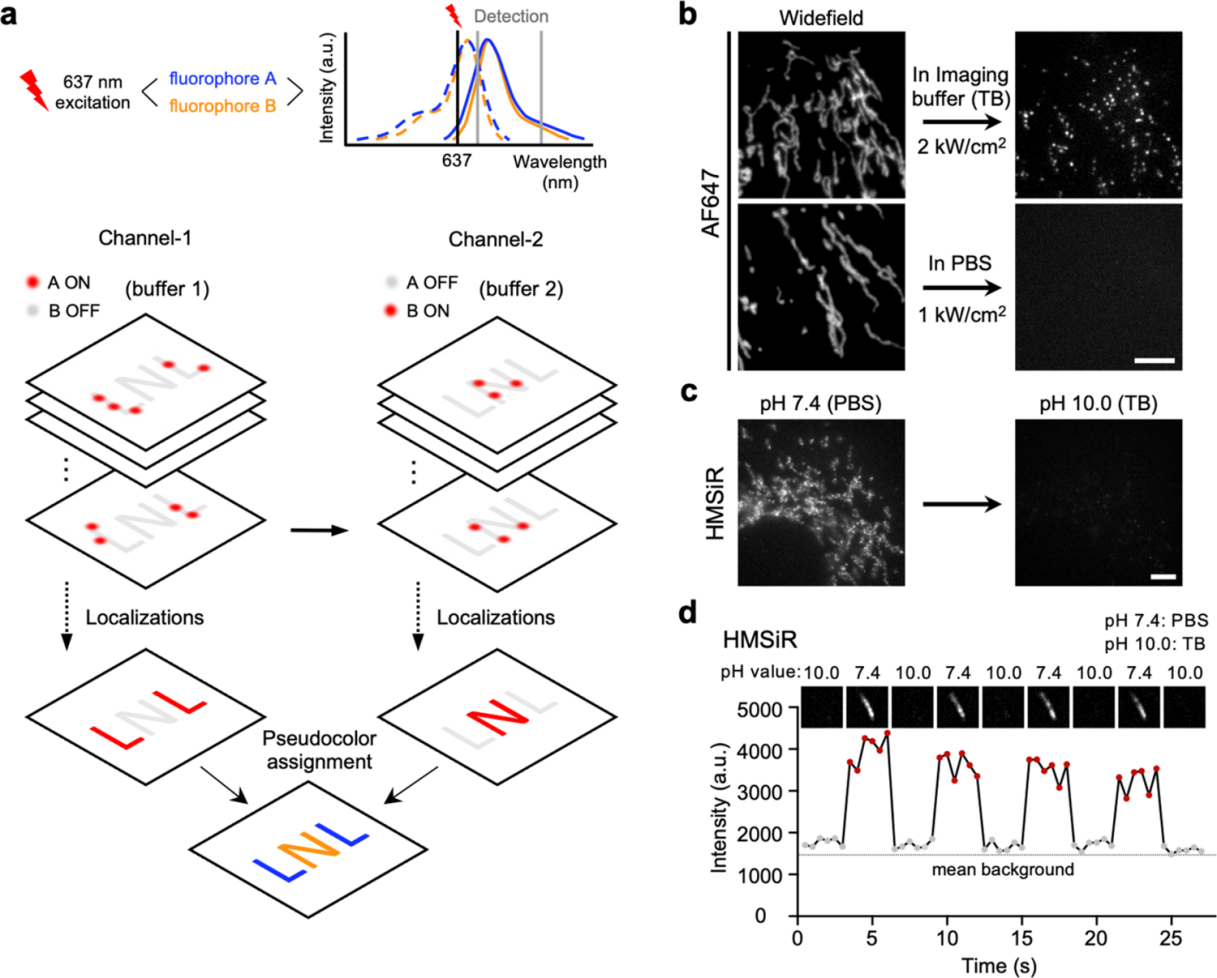
Principle of beSTORM. (a) Schematic of multitarget beSTORM imaging. fluorophores A and B are excited using a single laser at 637 nm. In the first channel, fluorophore A exhibits single-molecule blinking (ON state) while fluorophore B is in the OFF state in the buffer 1. Conversely, for the second channel, fluorophore B experiences a photoblinking process while fluorophore A is in the dark state in the buffer 2. A multitarget SMLM image is achieved by separately localizing fluorophores A and B in their respective channels. (b) Fluorescence of AF647 under different imaging conditions. AF647 showed repeated photoswitching when imaged in an imaging buffer containing thiols (TB); however, rapid photobleaching of AF647 occurred in PBS. (c) An increase in the pH value of the imaging buffer led to a significant decrease in the fluorescent intensity of HMSiR. (d) Reversibility of HMSiR fluorescence. The fluorescent states of HMSiR can be manipulated by exposing them to different buffer solutions. Scale bars, 5 μm (b, c).

With beSTORM, we present a simple method for multitarget STORM imaging, without relying on spectral properties. This method represents an intensity-based imaging methodology contributing an additional dimension for multitarget SMLM imaging. Our idea centers on distinguishing specific fluorophores sharing identical emission spectra by harnessing their distinct responsive blinking behaviors (i.e., ON and OFF states), which are influenced by their surroundings, rather than relying on their emission spectra. In our scheme, both fluorophores were excited using a single laser during sequential imaging (e.g., fluorophores A and B at 637 nm, Figure 1a). In the first channel, our aim was to maintain fluorophore A in an emissive state (ON state) while concurrently keeping fluorophore B in the OFF state. Conversely, within the second channel, the fluorescence emitting from fluorophore A was suppressed while simultaneously activating fluorophore B. Through appropriate manipulation of the fluorescent states of fluorophores A and B in both channels, we synergistically achieved dye separation within the same spectral emission, thus extending the capabilities of multitarget nanoscopic imaging.

To validate this idea, we started by considering the fluorescent dye, Alexa Fluor 647 (AF647), widely used for STORM imaging. AF647 exhibited robust blinking behavior (ON state) when imaged in a thiol-containing imaging buffer (TB), whereas it demonstrated significant photobleaching (OFF state) in phosphate buffered saline (PBS) (Figure 1b). The inherent characteristics of AF647 made it a candidate as a fluorophore for either buffer channel in our framework (fluorophore A, Figure 1a). Based on this, we proposed the buffer-exchange STORM imaging protocol, termed beSTORM. Subsequently, we searched for the second potential fluorescent dye (fluorophore B) that must be well-suited for localization imaging in PBS without thiol. In this pursuit, we used HMSiR, a commercially available red-absorbing Si-rhodamine dye exhibiting spontaneously photoblinking in PBS.^32^ Furthermore, the blinking behaviors, absorbance, and emission of HMSiR, could be adjusted by modifying the pH conditions.^32^ Notably, an increase in the pH value of the buffer solution resulted in a substantial reduction in both the fluorescence intensity and photoblinking of HMSiR (Figures 1c and S1). Importantly, these changes are reversible by substituting with buffer solutions at appropriate pH values (Figure 1d). Therefore, by employing HMSiR as fluorophore B, we can initially maintain HMSiR effectively in the OFF state by raising the pH value of the first buffer solution, and then reactivate HMSiR (ON state) for SMLM imaging by replacing the buffer with PBS (pH 7.4).

Overall, in the first channel, we conducted STORM imaging on specimens in TB with a pH of 10.0. This pH level was chosen to preserve the photostability of AF647, allowing us to specifically detect signals emitted by AF647 while simultaneously suppressing the single-molecule blinking of HMSiR and decreasing its fluorescence intensity. Subsequently, for the second channel, we replaced the TB with PBS at pH 7.4 to exclusively capture the HMSiR signals. In such manner, beSTORM suggests a multitarget nanoscopic strategy achieved through a simple buffer exchange.

### Performance of beSTORM with AF647 and HMSiR

**Figure 2:**
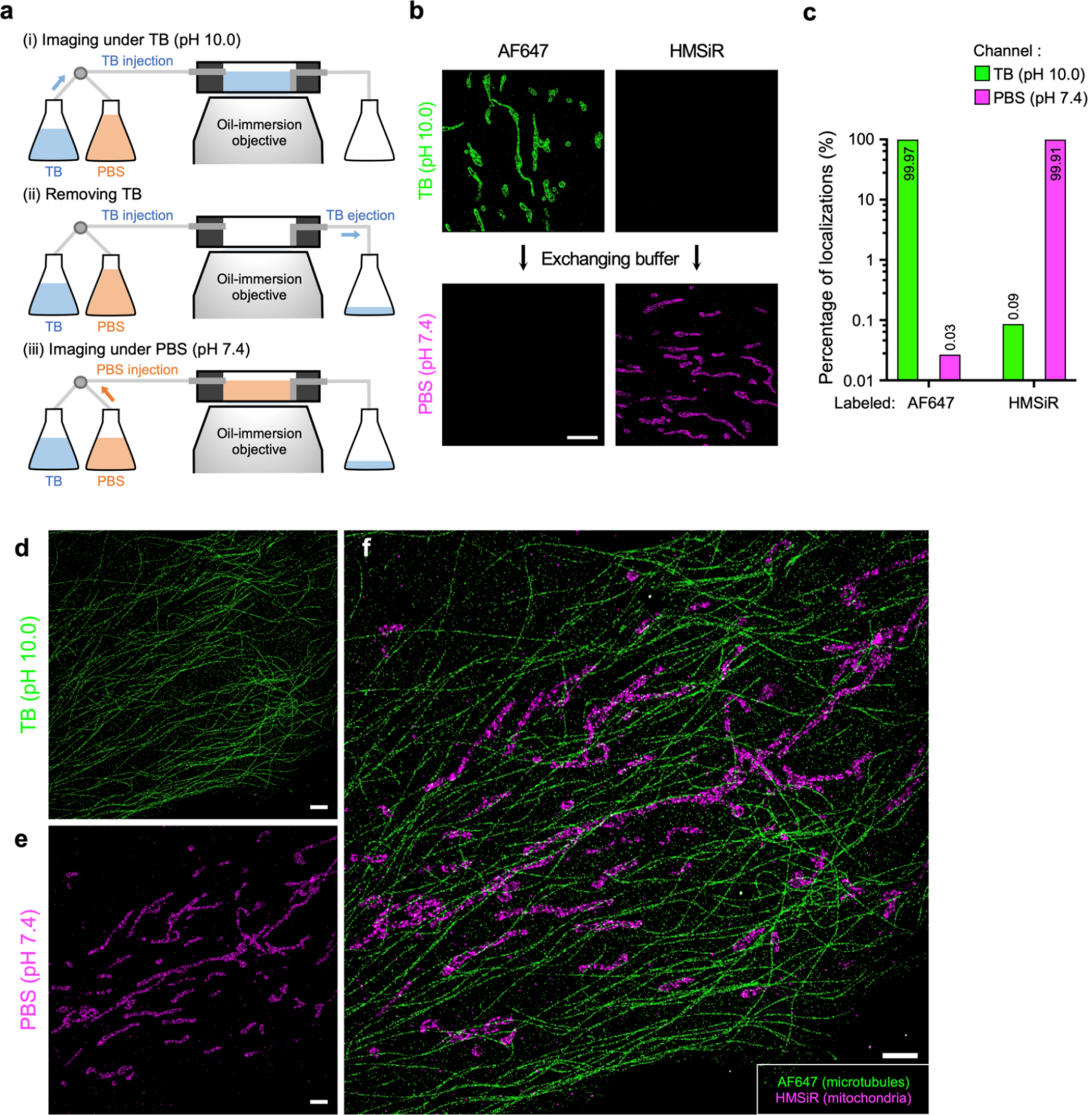
beSTORM imaging with far-red dyes. (a) Schematic of the beSTORM setup and imaging procedures using a simple sample chamber with a one-way inlet and outlet. The beSTORM involves directly replacing different buffer conditions, as shown in steps (i) to (iii). (b) beSTORM using AF647 and HMSiR dyes. Two dyes specifically labeled for the outer mitochondrial membrane of RPE-1 cells were separately tested in two different buffer channels. AF647 was only detectable in the TB solution, while HMSiR was exclusively visible in PBS upon intense illumination. (c) Leakage fraction of localizations obtained from (b), indicating an extremely low level of crosstalk between the two buffer channels. (d–f) Dual-target beSTORM images showing AF647-labeled microtubules and HMSiR-labeled mitochondria in an RPE-1 cell. Individual images reveal distinct cellular organelles (d, e). Scale bars, 2 μm (b, d–f).

In the context of beSTORM, a simple imaging chamber capable of exchanging buffers proves sufficient for multitarget SMLM imaging. The schematic setup for beSTORM is illustrated in Figure 2a. We opted for a sample chamber equipped with a one-way inlet and a one-way outlet (Figure S2). To minimize the bleed-through from AF647 to HMSiR, we conducted an extra prephotobleaching step before imaging HMSiR to fully extinguish AF647 signals following TB channel imaging. In the prephotobleaching step, a buffer with high pH is used as it creates a protective environment for HMSiR under high-power laser irradiation (Figures S1 and S3). Therefore, we replaced the TB (pH 10.0) with TN buffer (without thiols) at pH 11 for this process. Once AF647 signals were no longer detectable, we subsequently exchanged the buffer with PBS for HMSiR imaging. Our results revealed no discernible AF647 signals were observed in the PBS channel (Figure 2b). Similarly, the sample immunostained with HMSiR showed merely ∼0.1% localizations ratio (*N*_TB_: *N*_TB_ + *N*_PBS_, Figure 2b,c), suggesting negligible crosstalk of HMSiR detected in the TB channel. Our localization analysis revealed a crosstalk level of less than 0.1% between the two channels (Figure 2c).

To demonstrate the capability of beSTORM, we selected the dye combination of AF647 and HMSiR for labeling microtubules and mitochondrial membrane, respectively. Due to the significant overlap in the emission spectra of the two dyes (emission peak: 670 nm for AF647 and 669 nm for HMSiR), it enabled nearly chromatic aberration-free two-target SMLM imaging. With beSTORM, we noticed that the cellular images from each channel closely resembled those obtained by staining alone, exhibiting no apparent crosstalk or misidentification (Figure 2d,e). Hence, beSTORM enabled the isolation of the detection of either dye in its emissive state, resulting in the successful reconstruction of a two-target SMLM image (Figure 2f).

### Dye Combinations for Red-Emitting beSTORM

Within the framework of beSTORM, a method that exploits the buffer dimension in multitarget SMLM has been proposed to differentiate chromophores sharing the same emission spectra. It enables dual-target SMLM imaging (1 in TB + 1 in PBS, 1 + 1) using a single laser excitation (Figure 2f). Furthermore, multiplexing with different spectral channels (color dimension) brings an opportunity to double the available channels for multitarget SMLM. Hence, we embarked on developing a multiplexed beSTORM system that spans spectrally from far-red-emitting (637 nm laser excitation) to red-emitting (561 nm laser excitation) channels, aiming to achieve four-target beSTORM (2 + 2 using two excitation lasers). We specifically employed Dyomics 654 (Dy654) in combination with HMSiR as the far-red set (1 + 1) to diminish bleed-through between the far-red and red channels.^33−35^ It appears that Dy654-HMSiR showed comparable dual-target imaging while exhibiting low crosstalk (Figure S4).

**Figure 3:**
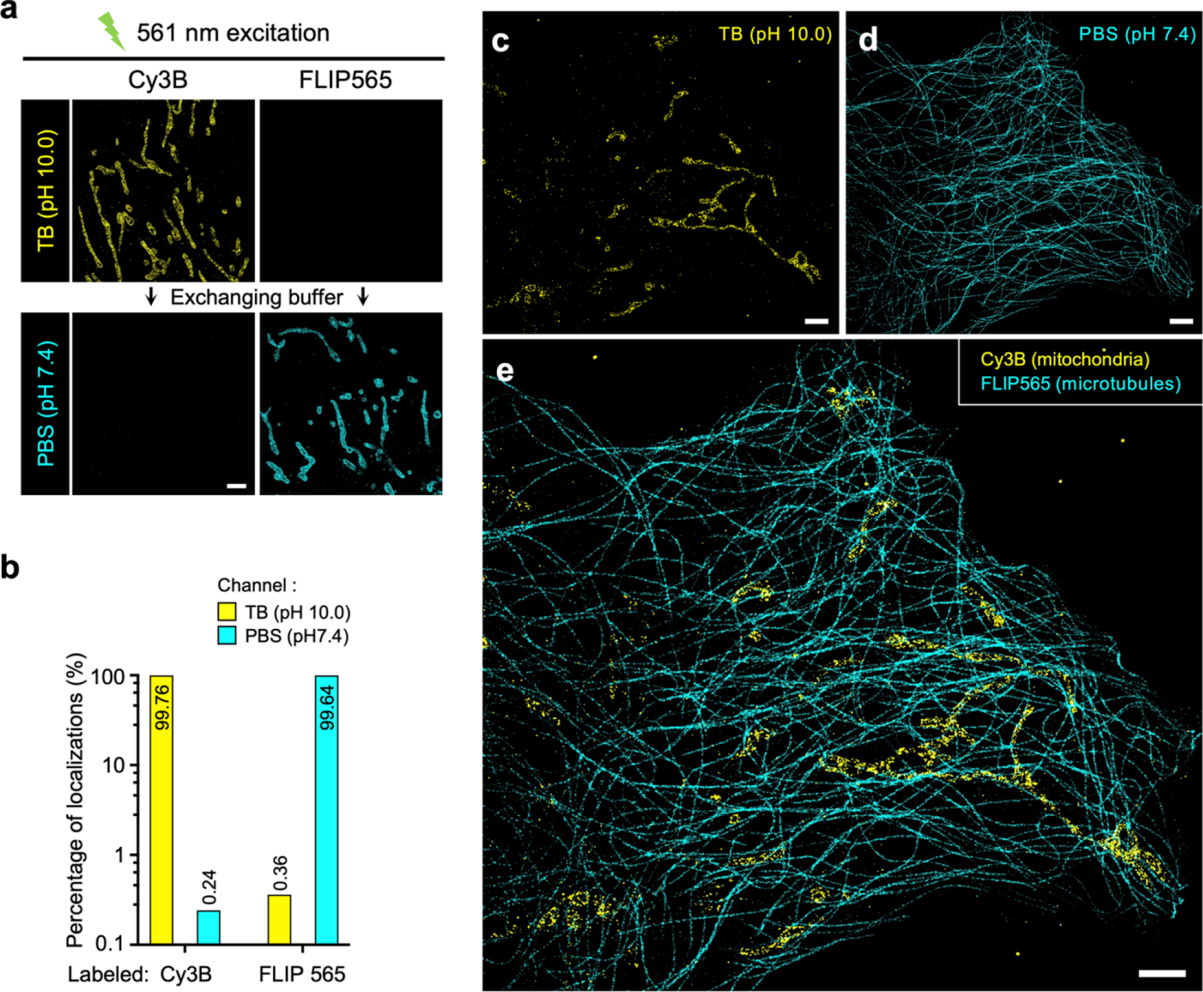
beSTORM validation for red-emitting dyes. (a–e) beSTORM utilizing red-emitting dyes (561 nm laser excitation). (a, b) Crosstalk assessment of the red-emitting dye pair, Cy3B and FLIP565. The outer mitochondrial membrane of RPE-1 cells was immunolabeled with either dye and examined separately in distinct beSTORM channels (a). The quantification of localization from (a) demonstrated minimal crosstalk between the two dyes (b). (c, d) beSTORM images acquired in different buffer channels revealing evident features of microtubules and mitochondrial membrane in a cell. (e) Composite beSTORM image from results (c, d). Scale bars, 2 μm (a, c–e).

Regarding red-emitting dyes, we included Cy3B as a candidate (fluorophore A) due to its favorable photostability in TB and significant fluorescent extinguishing in PBS (Table S1). For fluorophore B, we opted for FLIP565, another spontaneously blinking dye, whose fluorescent properties could also be manipulated by adjusting pH conditions^36^ (Figure S5). Therefore, the pair, Cy3B and FLIP565 was selected for red-emitting beSTORM imaging. Following the beSTORM procedure, when either dye was stained alone, our results showed unnoticeable localizations of Cy3B and FLIP565 detected in the unintended channels, namely, Cy3B in the PBS channel and FLIP565 in the TB channel, in line with our expectations (Figure 3a). Further quantitative analysis demonstrated negligible crosstalk, measuring less than 0.4% between two channels (Figure 3b). The beSTORM images presented anticipated cellular features, revealing distinct characteristics of microtubules and mitochondrial membranes in their corresponding red-emitting channels (Figure 3c–e).

### Multiplexed beSTORM Enables Simple Four-Target SMLM Imaging

**Figure 4:**
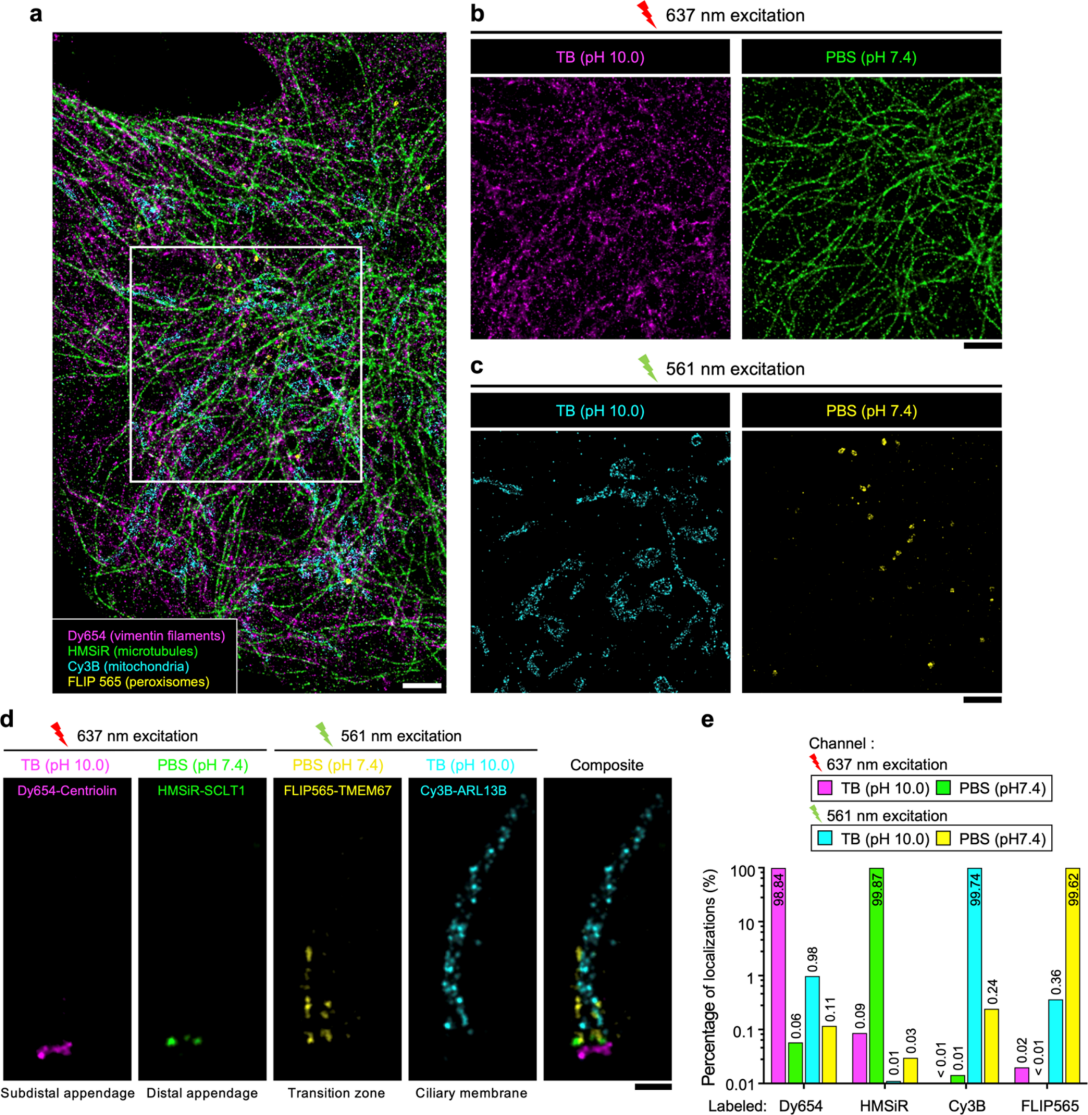
Multiplexed beSTORM imaging. (a) Reconstruction of a four-target localization image using beSTORM with dyes spanning red to far-red emission. Dy654, HMSiR, Cy3B, and FLIP565 were used to respectively label vimentin filaments, microtubules, mitochondria, and peroxisomes in an RPE-1 cell. (b, c) Recording specific cellular molecules in each beSTORM channel demonstrating the capability for precise differentiation of the four dyes within the far-red (b) and red (c) emission spectra. (d) beSTORM revealing the exclusive ciliary compartments of a mammalian primary cilium, highlighting distinct protein localizations of the subdistal appendage (Centriolin), distal appendage (SCLT1), ciliary membrane (ARL13B), and transition zone (TMEM67). (e) Localization analyses indicating low crosstalk fractions of less than 1% across all four channels. Scale bars, 500 nm (a–d).

By including far-red and red-emitting dyes, it enables multiplexed beSTORM for extended multicolor nanoscopy imaging. This streamlines the four-target SMLM protocol with two excitation lasers through a simple buffer exchange tactic (Figure S6). Here, we costained intermediate filaments, microtubules, the outer mitochondrial membrane, and peroxisomes with Dy654, HMSiR, Cy3B, and FLIP565, respectively, and conducted four-target SMLM imaging within red/far-red emission. Our beSTORM demonstrates successful reconstruction of a four-target nanoscopic image (Figure 4a), revealing distinguishable cellular features in their respective channels (Figure 4b,c). These results showcase the capability of beSTORM with precise differentiation of these four dyes within two chromatic channels. Subsequently, we further investigated subcellular structures within organelles using beSTORM capable of four-target imaging. We attempted to study the primary cilium, a densely packed organelle comprising several specific compartments that are technically challenging to resolve using conventional fluorescence microscopy. Again, Dy654, HMSiR, FLIP565, and Cy3B were used to label different compartments of a primary cilium, including the subdistal appendage, distal appendage, transition zone, and ciliary membrane. Significantly, the results indicated unequivocal identification of all four compartments in the reconstructed beSTORM images (Figure 4d). Our quantitative analyses report a mean localization precision of 10.65 nm (Figure S7) and crosstalk of less than 1% across all four channels (Figures 4e and S8). Together, we have presented a straightforward approach to accomplish proven four-target SMLM imaging by multiplexing two chromatic channels with beSTORM.

### Extended Multitarget Molecular-Resolution Imaging by Expansion beSTORM (Ex-beSTORM)

**Figure 5:**
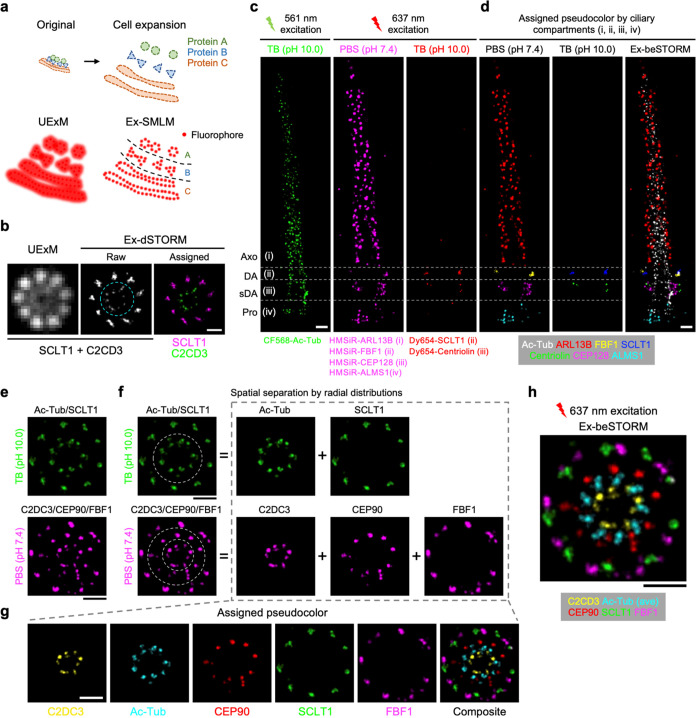
Extended multitarget molecular resolution imaging with expansion beSTORM. (a) Illustration depicting the localization-guided multitarget imaging of precharacterized cellular structures with Ex-SMLM, enabling molecular-level spatial identification of distinct proteins labeled with identical fluorophores. (b) Ex-dSTORM pinpointing colabeled SCLT1 and C2CD3, two centriolar proteins, as indicated by their radial distributions (dotted circle). (c) Seven-target Ex-beSTORM imaging of a primary cilium demonstrating four HMSiR-labeled proteins in the PBS channel and two Dy654-labeled proteins in the TB channel localized within distinct ciliary compartments: (i) Axo, axoneme; (ii) DA, distal appendage; (iii) sDA, subdistal appendage; (iv) Pro, proximal end. Additionally, the ciliary marker (Ac-Tub) was labeled with CF568. (d) Pseudocolored Ex-beSTORM results of the images from (c) revealing specific localizations of those proteins. (e) Ex-beSTORM imaging of two centriolar proteins (Ac-Tub and SCLT1, labeled with AF647) and three proteins (C2CD3, CEP90, and FBF1, labeled with HMSiR) in the TB and PBS channels, respectively. The result highlights nearly concentric 9-fold symmetric patterns with discernible radial arrangements. (f) Spatial partition for the images in (e) based on predetermined molecular distributions (dotted circles). (g) Five-target Ex-beSTORM imaging reconstruction of proteins spanning from the inner centriole wall to the outer DA, indicated by pseudocolor assignments. (h) Rotational averaging performed on signals from the centriole marker (Ac-Tub) in Ex-beSTORM, enhancing clarity in observing relative spatial relationships among proteins against the centriole. Scale bars, 500 nm (b–h).

Recent advances in cell expansion,^30,31^ achieved through the physically swelling biological samples, have been employed to facilitate superresolved imaging with conventional fluorescence microscopy. The increased spacing between protein complexes allows for a more accurate depiction of specific molecular arrangements or patterns. Lately, expansion SMLM (Ex-SMLM) further pushes the resolution limit, enabling fluorophores to be spatially isolated into concentrated clusters at the molecular level.^35,37^ In this work, we adroitly utilized the combined method to identify various proteins marked with the same fluorophores. This breakthrough extends the capacity to accommodate protein targets by simultaneously observing distinct, known biological structures (Figure 5a). Special attention is directed to centrioles, where two proteins, SCLT1 and C2CD3, colabeled with AF647, were distinctly identified according to their radial distributions using Ex-dSTORM (Figure 5b). Hence, we proceeded cell expansion for beSTORM (Ex-beSTORM) imaging; this allows for the visualization of multiple targets in a single channel and multiple buffer channels using a single laser for extended multitarget molecular-resolution imaging.

To substantiate this concept, we examined a primary cilium from a lateral view, where various structural compartments serve as an ideal spatial indicator for protein characterization. Herein, we immunolabeled six ciliary proteins using Dy654 or HMSiR, including the proximal end (Pro, ALMS1-HMSiR), subdistal appendage (sDA, CEP128-HMSiR and Centriolin-Dy654), distal appendage (DA, FBF1-HMSiR and SCLT1-Dy654), and axoneme (Axo, Arl13b-HMSiR). Additionally, we labeled the ciliary marker (Ac-Tub) with CF568 to delineate the profile of primary cilium. The far-red Ex-beSTORM images revealed distinctive features of six proteins located within predefined ciliary compartments ((i–iv), Figure 5c), with four in the PBS channel and two in the TB channel. Upon assigning pseudocolors to their respective compartments and channels, the reconstructed result displayed an illustrative seven-target Ex-dSTORM image of primary cilium (Figure 5d).

Likewise, most of the DA and its associated proteins are arranged in 9-fold symmetric distributions with varied radial dimensions,^35,38,39^ suggesting yet another advantageous spatial characteristic for protein discrimination. Considering their distinct radial outlines, we stained two proteins (Ac-Tub and SCLT1) with AF647 for the TB channel and three proteins (C2CD3, CEP90, and FBF1) with HMSiR for the PBS channel. The resulting Ex-beSTORM images successfully resolved proteins in both channels, unveiling nearly concentric 9-fold symmetric patterns with discernible radial arrangements (Figure 5e). This helped the segmentation of colabeled clusters based on their predetermined diameters (Figure 5f). By applying pseudocolors to individual targets initially labeled in the same color, Ex-beSTORM effectually generate a five-target image with molecular precision, depicting the proteins extending from the inner centriole wall to the outer DA (Figure 5g). Lastly, we performed rotational averaging to enhance the chirality of the centriole marker (Ac-Tub) for better observation of their relative spatial relationships with respect to the centriole (Figure 5h). Significantly, the multitarget image obtained from a single-round acquisition enables a comprehensive characterization of intact relative protein localizations within the same centriole. This is particularly challenging with the previous method, which necessitated averaging data from multiple rounds of two-color Ex-dSTORM imaging^35^ (Figure S9).

As an advancement in multiplexed nanoscopy, beSTORM provides an additional dimension for multitarget SMLM. By implementing buffer exchanges, we successfully differentiated between various fluorophores, even those with identical spectra, through strategic manipulation of their photoswitching properties, including fluorescent emission and blinking behaviors. Our initial demonstrations showcased dual-target SMLM imaging within the buffer dimension. Furthermore, we proposed a pragmatic solution for four-target SMLM imaging, seamlessly integrating with the color dimension, and achieving minimal crosstalk—less than 1% across all channels. beSTORM only necessitates a simple imaging chamber capable of buffer exchanging. Moreover, we conducted Ex-beSTORM imaging of colabeled cellular structures that became differentiable at the molecular level while capitalizing on the enhanced spatial resolution attained through the expansion process. This method has demonstrated ultraresolved distinction of up to six proteins within a single emission color, thereby avoiding chromatic aberration.

Beyond the results obtained in this study, beSTORM has manifested the potential to further diversify its color palette. One avenue involves incorporating green-emitting, spontaneously blinking fluorophores^40^ into the established beSTORM system to implement a direct six-target (3 + 3) SMLM imaging. Another promising direction is integrating beSTORM with other orthogonal methods, such as ratiometric, fluorescence-lifetime and spectrally resolved SMLM. Due to the inherent simplicity and compatibility of beSTORM, it has the potential to be seamlessly combined with those methods, expanding the accommodation of targets in both TB and PBS channels. One possible mixture may attain six-target (4 + 2) SMLM imaging under single laser excitation. This would target four dyes in the TB channel through spectrally resolved analysis^18^ and two dyes in PBS through either spectrally resolved or ratiometric analysis.^41^ Other advantageous extension of beSTORM is to increase the number of buffer channels. One viable approach could exploit additional pH-dependent channels on the use of spontaneously blinking dyes with a lower equilibrium constant (p*K*_cycl_) within the current beSTORM scheme. For instance, performing beSTORM imaging across a spectrum of solutions from alkaline and neutral to acidic conditions may enable multitarget SMLM imaging, spanning from (1 + 1 + 1) to (*n*_alk_ + *n*_neu_ + *n*_aci_). This can be implemented using a single laser either independently or in conjunction with other methodologies. Notably, besides HMSiR and FLIP565, which are specifically imaged under physiological conditions, numerous other self-blinking dyes working at various p*K*_cycl_ values have been accessible,^32,40,41^ thus making a further expansion of multichannel beSTORM possible.

Notably, since beSTORM involves buffer switching between image channels, it is advisable to preliminarily evaluate whether drastic changes in buffer conditions, such as pH levels, might cause alterations in cellular structures. As for its potential concern like labeling efficiency or sample integrity, our beSTORM data reported minimal impacts on the DA architecture when samples were exposed to a high pH buffer solution. Specifically, our five-target Ex-beSTORM data (Figure S9) provides structural interpretations of the DA configuration that match our earlier findings^35^ at the molecular level. Nevertheless, previous studies suggest that cytoskeleton systems, such as vimentin filaments, may become unstable at high pH levels.^42^ While our results do not indicate an obvious influence on the beSTORM images, we still cannot entirely rule out this issue arising from drastic pH variations. Therefore, a preliminary test before conducting beSTORM imaging procedures is recommended.

Furthermore, the positional misalignment between channels may occur after buffer-exchange steps as a consequence of uncertain mechanical shifts. To handle this situation, we utilized fiducial markers, such as microspheres, to align the channels with the same laser excitation (dual-target beSTORM). We calibrated the two buffer channels by jointly correcting their lateral drift and then localizing them separately for the final resulting images. This is feasible because the dyes and microspheres share the same emission wavelength. Typically, the lateral shift introduced during buffer-exchange processes in our beSTORM experiments was within a few micrometers, making it relatively simple to align the channels. For Ex-beSTORM, we employed the same strategy, except that a marker protein was used as the fiducial marker for in situ drift correction.

## Conclusions

Overall, beSTORM offers a straightforward solution for multiplexed SMLM imaging. All materials adopted in this study are commercially available, ensuring easy implementation for laboratories engaged in the development of SMLM. Besides, any fluorophores or buffer conditions meeting our beSTORM criteria could be flexibly customized for further applications. Moreover, beSTORM holds great promise for advancing multiplexed nanoscopy, allowing for versatile exploration. Each direction can be further combined to extend the range of accommodated targets. We are optimistic that the ease of implementation and the high potential compatibility with other modalities will render our method accessible for a wide range of applications, facilitating the investigation of complex biological systems with nanoscale precision.

## Methods/Experimental

### Optical Setup

Fluorescence imaging was performed on a modified optical configuration built on a commercial inverted microscope (Eclipse Ti2-E, Nikon) with a Nikon Perfect Focus System and a laser merge module (ILE, Spectral Applied Research) with individual controllers for four light sources. Beams from a 637 nm laser (OBIS 637 LX 140 mW, Coherent), a 561 nm laser (Jive 561 150 mW, Cobolt), a 488 nm laser (OPSL 488 LX 150 mW, Coherent), and a 405 nm laser (OBIS 405 LX 100 mW, Coherent) were homogenized (Borealis Conditioning Unit, Spectral Applied Research) and then focused onto the back focal plane of an oil-immersing objective (100 × 1.49, CFI Apo TIRF, Nikon) for wide-field illumination of samples. Fluorescent signals were spectrally filtered using emission filters (700/75, Chroma, 605/52, Chroma, Bellows Falls, VT) and captured with an electron-multiplying charge-coupled device (EMCCD) camera (iXon Life 888, Andor Technology) with an overall pixel size of 83.5 nm.

### Cell Culture

Human retinal pigment epithelial cells (hTERT RPE-1, ATCC-CRL-4000) were grown on coverslips coated with poly-l-lysine in Dulbecco’s modified Eagle’s medium (DMEM)/F-12 mixture medium supplemented with l-glutamine, HEPES (1:1; 11330–032, Gibco, Thermo Fisher Scientific), 10% fetal bovine serum (FBS, SH3010903, Hyclone), sodium bicarbonate (NaHCO_3_, S6014, Sigma-Aldrich), and 1% penicillin-streptomycin at 37 °C in a 5% CO_2_ environment. Primary cilium formation was initiated through a 24 h period of serum starvation.

### Antibodies

Detailed information regarding the primary antibodies employed in this study can be found in Immunostaining section and Table S2. The secondary antibodies included Alexa Fluor 647 (AF647, goat antimouse IgG1 A21240, goat antimouse IgG2b A21242, donkey antirabbit A31573, goat antirat A21247, Invitrogen), HMSiR (goat antimouse A202–01, goat antirabbit A204–01, goat antirat A203–01, Goryo Chemical), FLIP565 (goat antimouse 2–0002–202–4, goat antirabbit 2–0012–202–1, Abberior), and Alexa Fluor 488 (AF488, goat antimouse IgG2b A21141, Invitrogen). Distinct unlabeled secondary antibodies (goat antimouse IgG1 115–005–205, goat antimouse IgG2a 115–005–206, goat antimouse IgG2b 115–005–207, donkey antichicken IgY 703–005–155, donkey antirat 712–005–153, donkey antirabbit 711–005–152, Jackson ImmunoResearch) were conjugated with Dy654 *N*-hydroxysuccinimidyl (NHS) ester (654–01, Dyomics), CF568-NHS ester (92131, Biotium) or Cy3B-NHS ester (PA63100, Cytiva). The reaction was conducted in 0.1 M NaHCO_3_ in the dark for 30 min. Labeled antibodies were purified by gel filtration using NAP-5 columns (17085301, Cytiva) according to the manufacturer’s instructions. The corresponding dilution ratio of the labeled secondary antibody for each sample can be found in Immunostaining section.

### Buffer Solutions for beSTORM

The beSTORM imaging procedure utilized buffers, including thiol-containing buffer (TB), TN buffer at pH 11, and PBS. The TB consisted of 50 mM Tris (pH 10.0, T1503, Sigma-Aldrich), 10 mM sodium chloride (NaCl, 31434, Sigma-Aldrich), 10 mM β-mercaptoethylamine (30070, Sigma-Aldrich), 10% glucose (G5767, Sigma-Aldrich), 0.5 mg mL^–1^ glucose oxidase (G2133, Sigma-Aldrich), and 40 μg mL^–1^ catalase (C9322, Sigma-Aldrich). The TN buffer at pH 11 was composed of 50 mM Tris and 10 mM NaCl.

### Expanded Sample Preparation (UExM)

Before fixation, RPE-1 cells on 12 mm coverslips were deprived of serum for 24 h to induce cilium formation. Cells were fixed with either 4% PFA at room temperature (RT) for the seven-target experiment or ice-cold methanol at −20 °C for the five-target experiment for 10 min, followed by incubation in a perfusion solution containing 1.4% PFA and 2% acrylamide (AA, A4058, Sigma-Aldrich) in PBS for 5 h at 37 °C. Next, the gelation solution containing 19% (w/w) sodium acrylate (SA, 408220, Sigma-Aldrich), 10% (w/w) AA, 0.1% (w/w) bis-acrylamide (BIS, M1533, Sigma-Aldrich), 0.5% (w/w) *N*,*N*,*N*′,*N*′-tetramethylethylenediamine (TEMED, 1610801, Bio-Rad), and 0.5% (w/w) ammonium persulfate (APS, 1610700, Bio-Rad) was added to the perfused cells in a chamber on ice for 3 min, followed by a 1 h incubation at 37 °C for polymerization. Subsequently, coverslips with hydrogel were incubated in fresh denaturation buffer consisting of 200 mM sodium dodecyl sulfate (SDS, 0227, VWR Life science), 200 mM sodium chloride (NaCl, 31434, Sigma-Aldrich) in 50 mM Tris (pH 8.8, J831, VWR Life science) for 15 min at RT with gentle shaking. The hydrogels were then boiled at 95 °C in the denaturation buffer for 1.5 h. After denaturation, hydrogels with denatured samples were processed for the first expansion in ddH_2_O overnight. Next, the expanded gels were washed with PBS and kept in PBS before immunostaining (details in the next section). After staining, the hydrogels then expanded to their maximal size until ddH_2_O replacement completed. To retain the size of the expanded hydrogel in various buffers, a neutral acrylamide gel was cross-linked onto the expanded hydrogel, chemically binding it to bind-silane-treated coverslips. The expanded hydrogels were incubated twice in a freshly prepared re-embedding solution (10% (w/w) AA, 0.15% (w/w) BIS, 0.05% (w/w) TEMED, 0.05% (w/w) APS in ddH_2_O) for 25 min each time at RT with gentle shaking. The coverslips were washed with ddH_2_O and absolute ethanol (32221, Sigma-Aldrich), and coated in a freshly prepared working solution containing 5 μL bind-silane (abx082155, Abbexa), 8 mL absolute ethanol, 200 μL acetic acid (33209, Sigma-Aldrich), and 1.8 mL ddH_2_O. The coverslips were then washed with absolute ethanol and allowed to air-dry. Subsequently, the expanded hydrogel, filled with the re-embedding solution, was carefully transferred onto bind-silane-treated coverslips, with excess solution removed from the hydrogels using laboratory wipes. Another untreated coverslip was placed on top of the hydrogels for the subsequent polymerization process. This procedure was performed in a nitrogen-filled humidified chamber at 37 °C for 2 h. Following polymerization, the re-embedding gels underwent three washes in ddH_2_O, each lasting 20 min, and kept in PBS before imaging.

### Immunostaining

#### Far-Red Emitting Dual-Target beSTORM

Cells on 18 mm coverslips were fixed with 3% paraformaldehyde (PFA; 16%, 15710, Electron Microscopy Sciences) and 0.1% glutaraldehyde (GA; 8%, 16020, Electron Microscopy Sciences) at RT in 1× phosphate-buffered saline (PBS; diluted from 10× PBS, K813, VWR Life science) for 10 min. After wash with PBS, cells were then permeabilized with 0.1% Triton X-100 (T8787, Sigma-Aldrich) in PBS (0.1% PBST) for 10 min and blocked with blocking buffer (3% bovine serum albumin (BSA, A9647, Sigma-Aldrich) in 0.1% PBST) for 30 min. Primary antibodies, rat anti-α-tubulin (1:100 dilution, ab6160, Abcam) and rabbit anti-TOMM20 (1:250 dilution, ab186735, Abcam), were diluted in blocking buffer and incubated with samples at RT for 1 h. Cells were washed five times with 0.1% PBST to remove unbound primary antibodies. Subsequently, cells were stained with secondary antibodies labeled with AF647 (1:200 dilution, antirat), Dy654 (1:100 dilution, antirat), and HMSiR (1:200 dilution, antirabbit). After five-time washes, cells were stored in PBS at 4 °C.

#### Red Emitting Dual-Target beSTORM

Cells on 18 mm coverslips were treated with the same fixation, permeabilization, blocking, immunostaining processes as described in the preceding section. The primary bodies used were mouse anti-α-tubulin (1:250 dilution, sc32293, Santa Cruz) and rabbit anti-TOMM20 (1:250 dilution). Secondary antibodies labeled with Cy3B (1:100 dilution, antirabbit) and FLIP565 (1:200 dilution, antimouse) were used to label primary antibodies.

#### Four-Target beSTORM

Cells on 18 mm coverslips were subjected to the identical fixation, permeabilization, blocking, immunostaining process as described above. The primary antibodies utilized consisted of chicken antivimentin (1:200 dilution, ab24525, Abcam), rat anti-α-tubulin (diluted at 1:250), rabbit anti-TOMM20 (diluted at 1:250), and mouse anti-PMP70 (1:100 dilution, SAB4200181, Sigma-Aldrich). For the labeling of these primary antibodies, secondary antibodies labeled with Dy654 (1:100 dilution, antichicken), HMSiR (1:200 dilution, antirat), Cy3B (1:100 dilution, antirabbit), and FLIP565 (1:200 dilution, antimouse) were employed.

#### Four-Target beSTORM of Primary Cilium

Prior to fixation, RPE-1 cells on 18 mm coverslips were serum-starved for 24 h to induce cilium formation. Subsequently, samples were fixed with ice-cold ethanol at −20 °C for 10 min. Following, the cells underwent the same permeabilization, blocking, immunostaining processes. The primary antibodies employed included mouse IgG1 anti-Centriolin (1:200 dilution, sc-365521, Santa Cruz), rat anti-SCLT1 (1:100 dilution, gift from Meng-Fu Bryan Tsou, Memorial Sloan Kettering Cancer Center), mouse IgG2a anti-ARL13B (1:500 dilution, ab136648, Abcam), and rabbit anti-TMEM67 (1:200 dilution, 13975–1-AP, Proteintech). Secondary antibodies labeled with Dy654 (1:100 dilution, antimouse IgG1), HMSiR (1:200 dilution, antirat), Cy3B (1:100 dilution, antimouse IgG2a), and FLIP565 (1:200 dilution, antirabbit) were utilized.

#### Seven-Target Ex-beSTORM of Primary Cilium

The hydrogels were stained with primary and secondary antibodies, diluted in 2% BSA/PBS at 37 °C in the 1.5 mL Eppendorf for 3 h with gentle shaking, followed by washing 3 times with 0.1% Tween 20 (P137, Sigma-Aldrich) in PBS and once with PBS for 20 min each. Subsequently, the hydrogels were expanded in ddH_2_O until reaching their maximal expansion via exchanging ddH_2_O at least 3 times. Finally, the expanded hydrogels underwent a re-embedding process and chemical binding onto the bind-silane-treated 18 mm coverslips. To achieve seven-target labeling, samples underwent two rounds of immunostaining. In the first-round labeling, primary antibodies included mouse IgG2b anti-Acetyl-α-Tubulin (Ac-Tub,1:500 dilution, 32–2700, Invitrogen), mouse IgG1 anti-Centriolin (1:100 dilution), rabbit anti-FBF1 (1:150 dilution,11531–1-AP, Proteintech), and rabbit anti-ARL13B (1:200 dilution,17711–1-AP, Proteintech). These primary antibodies were tagged by secondary antibodies labeled with Dy654 (1:100 dilution, antimouse IgG1), HMSiR (1:100 dilution, antirabbit), and CF568 (1:100 dilution, antimouse IgG2b). For the second round, primary antibodies including rat anti-SCLT1 (1:100 dilution), rabbit anti-CEP128 (1:200 dilution, ab118797, Abcam), rabbit anti-ALMS1 (1:500 dilution, A301–815A, Bethyl), and mouse IgG2b anti-ATP synthase (1:100 dilution, ab109867, Abcam) were applied. Secondary antibodies labeled with Dy654 (1:100 dilution, antirat), HMSiR (1:100 dilution, antirabbit), and AF488 (1:100 dilution, antimouse IgG2b) were utilized.

#### Five-Target Ex-beSTORM of Centriole

The samples also underwent two rounds of immunostaining. In the first round, primary antibodies included rat anti-SCLT1 (1:100 dilution), mouse IgG2b anti-Ac-Tub (1:500 dilution), rabbit anti-FBF1 (1:100 dilution), and rabbit anti-C2CD3 (1:100 dilution) were used. These primary antibodies were labeled using secondary antibodies tagged with AF647 (1:100 dilution, antimouse IgG2b and antirat) and HMSiR (1:100 dilution, antirabbit). In the second round, we employed rabbit anti-CEP90 (1:150 dilution, 14413–1-AP, Proteintech) and mouse IgG2b anti-ATP synthase (1:100) as primary antibodies labeled with HMSiR (1:100 dilution, antirabbit) and AF488 (1:100 dilution, antimouse IgG2b).

### beSTORM Imaging

For the buffer-exchange process, we adopted a commercial magnetic imaging chamber featuring a one-way inlet and a one-way outlet (CM-B18–1, Live Cell Instrument, Figure S2). The detailed schematic procedure of beSTORM is shown in the Figure S6. Briefly, the sequential imaging began with the recording of the far-red-emitting TB channel (in TB), followed by the acquisition of the far-red-emitting PBS channel (in PBS). Subsequently, the red-emitting TB channel was recorded, followed by the acquisition of the red-emitting PBS channel. During the TB channel acquisition, the 637 and 561 nm laser lines were operated at an intensity of ∼1.5–3 kW cm^–2^ to quench most of the fluorophores (AF647, Dy654, or Cy3B). A weak 405 nm beam was used to activate a portion of the dyes, converting them from a long-lived dark state to a ground state. After acquiring the TB channel, a prephotobleaching procedure was executed to minimize crosstalk between the TB and PBS channels. This involved substituting the imaging buffer with TN buffer at pH 11 and then subjecting the sample to intense irradiation with either 637 or 561 nm laser lines with 405 nm laser activation until the signals of AF647, Dy654, or Cy3B became nearly undetectable. Subsequently, the remaining TN buffer was replaced with PBS before imaging. For the PBS channels (HMSiR or FLIP565), the imaging laser at an intensity of 1–2 kW cm^–2^ for 637 nm or 3–5 kW cm^–2^ for 561 nm was applied without 405 nm laser activation. The collected single-molecule signals were cleaned by the corresponding emission filters and registered on an EMCCD. Typically, for each beSTORM image, 15,000–30,000 frames were acquired at a rate of 50 fps. The position of the individual single-molecule peak was then localized using MetaMorph Superresolution Module (Molecular Devices) based on a wavelet segmentation algorithm. The localization images were denoised with the Gaussian filter of 0.75–1 pixel.

### Ex-beSTORM Imaging

The setup and procedure for Ex-beSTORM imaging was adapted from the beSTORM protocol with modifications detailed below. The 488 nm laser line was introduced and intermittently switched on every 800 frames for in situ drift correction during which all fluorescent signals underwent a quad-band filter (ZET405/488/561/640 mv2, Chroma). The CF568 channel was captured with an additional short-pass filter (BSP01–633R-25, Semrock).

### Drift Correction and Image Registration

For beSTORM, 0.1 μm TetraSpeck microspheres (T7279, Invitrogen) were employed as fiducial markers to correct lateral drift. The position drift was measured during acquisition and corrected via frame-by-frame correlation of the markers using ImageJ. For Ex-beSTORM, in situ drift correction was implemented as previously reported.^35^ Briefly, the marker protein (ATP synthase, 1/100 dilution, ab109867, Abcam) was initially labeled with Alexa Fluor 488 (1/100 dilution) before imaging. Lateral position drift (marker protein) was intermittently recorded during acquisition and compensated using a homemade algorithm. Sets of images with marker signals were eliminated before localizing each single-molecule peak. Chromatic aberration compensation was performed with a customized code that relocated each pixel of a red-emitting image to its corrected position, using a predefined correction function obtained through parabolic mapping of multiple calibration microspheres.

### Crosstalk and Localization Precision Analyses

To quantify the channel crosstalk in beSTORM imaging, we prepared samples labeled the outer mitochondrial membrane (anti-TOMM20) with AF647, Dy654, HMSiR, Cy3B, or FLIP565. Samples were tested following the beSTORM procedure, starting from the far-red-emitting TB channel, far-red-emitting PBS channel, red-emitting TB channel, to red-emitting PBS channel (Figure S6). The fraction of localizations contributing to crosstalk was calculated by dividing the number of localizations of the labeled fluorophore signal inside the undesired channel (such as the localizations of AF647 in far-red-emitting PBS channel) by the total number of localizations in all four channels (Figure S8). For crosstalk analysis between the TB and PBS channels within far-red or red channel alone, the number of localizations of the labeled fluorophore signal inside the undesired channel was divided by the total number of localizations in the corresponding TB and PBS channels. Localization precision of beSTORM images using different dyes were evaluated by nearest neighbor based analysis (NeNA).^43^ The achieved localization precisions are reported in Figure S7.

## Data Availability

All the data supporting the findings described this study are available within the article and Supporting Information and are available from the corresponding author upon reasonable request.

## Supporting Information Available

Effect of pH values on fluorescence intensity and photoblinking of the beSTORM fluorophores; imaging chamber; protective role of dyes under high-pH buffer; far-red beSTORM imaging; multitarget beSTORM procedures; localization precision assessment of beSTORM; crosstalk analysis of beSTORM; multitarget Ex-beSTORM; evaluation of red-emitting dyes on beSTORM, and antibodies list (PDF)

## Notes

**Reproducibility** All experiments were conducted at least 3 times, and representative images are shown for each experiment.

## References

[ref1] Lelek M.; Gyparaki M. T.; Beliu G.; Schueder F.; Griffié J.; Manley S.; Jungmann R.; Sauer M.; Lakadamyali M.; Zimmer C. Single-molecule localization microscopy. Nat. Rev. Methods Primers 2021, 1 (1), 39 10.1038/s43586-021-00038-x. 35663461 PMC9160414

[ref2] Betzig E.; Patterson G. H.; Sougrat R.; Lindwasser O. W.; Olenych S.; Bonifacino J. S.; Davidson M. W.; Lippincott-Schwartz J.; Hess H. F. Imaging intracellular fluorescent proteins at nanometer resolution. Science 2006, 313 (5793), 1642–1645. 10.1126/science.1127344. 16902090

[ref3] Hess S. T.; Girirajan T. P.; Mason M. D. Ultra-high resolution imaging by fluorescence photoactivation localization microscopy. Biophys. J. 2006, 91 (11), 4258–4272. 10.1529/biophysj.106.091116. 16980368 PMC1635685

[ref4] Rust M. J.; Bates M.; Zhuang X. Sub-diffraction-limit imaging by stochastic optical reconstruction microscopy (STORM). Nat. Methods 2006, 3 (10), 793–796. 10.1038/nmeth929. 16896339 PMC2700296

[ref5] Heilemann M.; Van De Linde S.; Schüttpelz M.; Kasper R.; Seefeldt B.; Mukherjee A.; Tinnefeld P.; Sauer M. Subdiffraction-resolution fluorescence imaging with conventional fluorescent probes. Angew. Chem., Int. Ed. 2008, 47 (33), 6172–6176. 10.1002/anie.200802376. 18646237

[ref6] Sharonov A.; Hochstrasser R. M. Wide-field subdiffraction imaging by accumulated binding of diffusing probes. Proc. Natl. Acad. Sci. U.S.A. 2006, 103 (50), 18911–18916. 10.1073/pnas.0609643104. 17142314 PMC1748151

[ref7] Jungmann R.; Steinhauer C.; Scheible M.; Kuzyk A.; Tinnefeld P.; Simmel F. C. Single-molecule kinetics and super-resolution microscopy by fluorescence imaging of transient binding on DNA origami. Nano Lett. 2010, 10 (11), 4756–4761. 10.1021/nl103427w. 20957983

[ref8] Sauer M.; Heilemann M. Single-Molecule Localization Microscopy in Eukaryotes. Chem. Rev. 2017, 117 (11), 7478–7509. 10.1021/acs.chemrev.6b00667. 28287710

[ref9] Sigal Y. M.; Zhou R.; Zhuang X. Visualizing and discovering cellular structures with super-resolution microscopy. Science 2018, 361 (6405), 880–887. 10.1126/science.aau1044. 30166485 PMC6535400

[ref10] Huang B.; Jones S. A.; Brandenburg B.; Zhuang X. Whole-cell 3D STORM reveals interactions between cellular structures with nanometer-scale resolution. Nat. Methods 2008, 5 (12), 1047–1052. 10.1038/nmeth.1274. 19029906 PMC2596623

[ref11] Shroff H.; Galbraith C. G.; Galbraith J. A.; White H.; Gillette J.; Olenych S.; Davidson M. W.; Betzig E. Dual-color superresolution imaging of genetically expressed probes within individual adhesion complexes. Proc. Natl. Acad. Sci. U.S.A. 2007, 104 (51), 20308–20313. 10.1073/pnas.0710517105. 18077327 PMC2154427

[ref12] Dempsey G. T.; Vaughan J. C.; Chen K. H.; Bates M.; Zhuang X. Evaluation of fluorophores for optimal performance in localization-based super-resolution imaging. Nat. Methods 2011, 8 (12), 1027–1036. 10.1038/nmeth.1768. 22056676 PMC3272503

[ref13] Pertsinidis A.; Zhang Y.; Chu S. Subnanometre single-molecule localization, registration and distance measurements. Nature 2010, 466 (7306), 647–651. 10.1038/nature09163. 20613725

[ref14] Bossi M.; Fölling J.; Belov V. N.; Boyarskiy V. P.; Medda R.; Egner A.; Eggeling C.; Schönle A.; Hell S. W. Multicolor far-field fluorescence nanoscopy through isolated detection of distinct molecular species. Nano Lett. 2008, 8 (8), 2463–2468. 10.1021/nl801471d. 18642961

[ref15] Lampe A.; Haucke V.; Sigrist S. J.; Heilemann M.; Schmoranzer J. Multi-colour direct STORM with red emitting carbocyanines. Biol. Cell 2012, 104 (4), 229–237. 10.1111/boc.201100011. 22187967

[ref16] Zhang Y.; Schroeder L. K.; Lessard M. D.; Kidd P.; Chung J.; Song Y.; Benedetti L.; Li Y.; Ries J.; Grimm J. B.; et al. Nanoscale subcellular architecture revealed by multicolor three-dimensional salvaged fluorescence imaging. Nat. Methods 2020, 17 (2), 225–231. 10.1038/s41592-019-0676-4. 31907447 PMC7028321

[ref17] Wu W.; Luo S.; Fan C.; Yang T.; Zhang S.; Meng W.; Xu T.; Ji W.; Gu L. Tetra-color superresolution microscopy based on excitation spectral demixing. Light: Sci. Appl. 2023, 12 (1), 9 10.1038/s41377-022-01054-6. 36588110 PMC9806106

[ref18] Zhang Z.; Kenny S. J.; Hauser M.; Li W.; Xu K. Ultrahigh-throughput single-molecule spectroscopy and spectrally resolved super-resolution microscopy. Nat. Methods 2015, 12 (10), 935–938. 10.1038/nmeth.3528. 26280329

[ref19] Song K.-H.; Zhang Y.; Brenner B.; Sun C.; Zhang H. F. Symmetrically dispersed spectroscopic single-molecule localization microscopy. Light: Sci. Appl. 2020, 9 (1), 92 10.1038/s41377-020-0333-9. 32509299 PMC7248114

[ref20] Thiele J. C.; Helmerich D. A.; Oleksiievets N.; Tsukanov R.; Butkevich E.; Sauer M.; Nevskyi O.; Enderlein Jr. Confocal fluorescence-lifetime single-molecule localization microscopy. ACS Nano 2020, 14 (10), 14190–14200. 10.1021/acsnano.0c07322. 33035050

[ref21] Oleksiievets N.; Mathew C.; Thiele J. C.; Gallea J. I.; Nevskyi O.; Gregor I.; Weber A.; Tsukanov R.; Enderlein Jr. Single-molecule fluorescence lifetime imaging using wide-field and confocal-laser scanning microscopy: A comparative analysis. Nano Lett. 2022, 22 (15), 6454–6461. 10.1021/acs.nanolett.2c01586. 35792810 PMC9373986

[ref22] Tam J.; Cordier G. A.; Borbely J. S.; Alvarez A. S.; Lakadamyali M. Cross-talk-free multi-color STORM imaging using a single fluorophore. PLoS One 2014, 9 (7), e101772 10.1371/journal.pone.0101772. 25000286 PMC4084994

[ref23] Valley C. C.; Liu S.; Lidke D. S.; Lidke K. A. Sequential superresolution imaging of multiple targets using a single fluorophore. PLoS One 2015, 10 (4), e0123941 10.1371/journal.pone.0123941. 25860558 PMC4393115

[ref24] Yi J.; Manna A.; Barr V. A.; Hong J.; Neuman K. C.; Samelson L. E. madSTORM: a superresolution technique for large-scale multiplexing at single-molecule accuracy. Mol. Biol. Cell 2016, 27 (22), 3591–3600. 10.1091/mbc.e16-05-0330. 27708141 PMC5221591

[ref25] Klevanski M.; Herrmannsdoerfer F.; Sass S.; Venkataramani V.; Heilemann M.; Kuner T. Automated highly multiplexed super-resolution imaging of protein nano-architecture in cells and tissues. Nat. Commun. 2020, 11 (1), 1552 10.1038/s41467-020-15362-1. 32214101 PMC7096454

[ref26] Jungmann R.; Avendaño M. S.; Woehrstein J. B.; Dai M.; Shih W. M.; Yin P. Multiplexed 3D cellular super-resolution imaging with DNA-PAINT and Exchange-PAINT. Nat. Methods 2014, 11 (3), 313–318. 10.1038/nmeth.2835. 24487583 PMC4153392

[ref27] Agasti S. S.; Wang Y.; Schueder F.; Sukumar A.; Jungmann R.; Yin P. DNA-barcoded labeling probes for highly multiplexed Exchange-PAINT imaging. Chem. Sci. 2017, 8 (4), 3080–3091. 10.1039/C6SC05420J. 28451377 PMC5380918

[ref28] Strauss S.; Jungmann R. Up to 100-fold speed-up and multiplexing in optimized DNA-PAINT. Nat. Methods 2020, 17 (8), 789–791. 10.1038/s41592-020-0869-x. 32601424 PMC7610413

[ref29] Schueder F.; Rivera-Molina F.; Su M.; Marin Z.; Kidd P.; Rothman J. E.; Toomre D.; Bewersdorf J. Unraveling cellular complexity with transient adapters in highly multiplexed super-resolution imaging. Cell 2024, 187 (7), 1769–1784.e18. 10.1016/j.cell.2024.02.033. 38552613 PMC12135969

[ref30] Chen F.; Tillberg P. W.; Boyden E. S. Expansion microscopy. Science 2015, 347 (6221), 543–548. 10.1126/science.1260088. 25592419 PMC4312537

[ref31] Gambarotto D.; Zwettler F. U.; Le Guennec M.; Schmidt-Cernohorska M.; Fortun D.; Borgers S.; Heine J.; Schloetel J.-G.; Reuss M.; Unser M.; et al. Imaging cellular ultrastructures using expansion microscopy (U-ExM). Nat. Methods 2019, 16 (1), 71–74. 10.1038/s41592-018-0238-1. 30559430 PMC6314451

[ref32] Uno S.-n.; Kamiya M.; Yoshihara T.; Sugawara K.; Okabe K.; Tarhan M. C.; Fujita H.; Funatsu T.; Okada Y.; Tobita S.; Urano Y. A spontaneously blinking fluorophore based on intramolecular spirocyclization for live-cell super-resolution imaging. Nat. Chem. 2014, 6 (8), 681–689. 10.1038/nchem.2002. 25054937

[ref33] Helmerich D. A.; Beliu G.; Matikonda S. S.; Schnermann M. J.; Sauer M. Photoblueing of organic dyes can cause artifacts in super-resolution microscopy. Nat. Methods 2021, 18 (3), 253–257. 10.1038/s41592-021-01061-2. 33633409 PMC10802917

[ref34] Stone M. B.; Veatch S. L. Far-Red Organic Fluorophores Contain a Fluorescent Impurity. ChemPhysChem 2014, 15 (11), 2240–2246. 10.1002/cphc.201402002. 24782148 PMC4180537

[ref35] Chang T.-J. B.; Hsu J. C.-C.; Yang T. T. Single-molecule localization microscopy reveals the ultrastructural constitution of distal appendages in expanded mammalian centrioles. Nat. Commun. 2023, 14 (1), 1688 10.1038/s41467-023-37342-x. 36973278 PMC10043031

[ref36] Belov V. N.; Bossi M. L.; Fölling J.; Boyarskiy V. P.; Hell S. W. Rhodamine spiroamides for multicolor single-molecule switching fluorescent nanoscopy. Chem. - Eur. J. 2009, 15 (41), 10762–10776. 10.1002/chem.200901333. 19760719

[ref37] Zwettler F. U.; Reinhard S.; Gambarotto D.; Bell T. D.; Hamel V.; Guichard P.; Sauer M. Molecular resolution imaging by post-labeling expansion single-molecule localization microscopy (Ex-SMLM). Nat. Commun. 2020, 11 (1), 3388 10.1038/s41467-020-17086-8. 32636396 PMC7340794

[ref38] Yang T. T.; Chong W. M.; Wang W.-J.; Mazo G.; Tanos B.; Chen Z.; Tran T. M. N.; Chen Y.-D.; Weng R. R.; Huang C.-E.; et al. Super-resolution architecture of mammalian centriole distal appendages reveals distinct blade and matrix functional components. Nat. Commun. 2018, 9 (1), 2023 10.1038/s41467-018-04469-1. 29789620 PMC5964178

[ref39] Bowler M.; Kong D.; Sun S.; Nanjundappa R.; Evans L.; Farmer V.; Holland A.; Mahjoub M. R.; Sui H.; Loncarek J. High-resolution characterization of centriole distal appendage morphology and dynamics by correlative STORM and electron microscopy. Nat. Commun. 2019, 10 (1), 993 10.1038/s41467-018-08216-4. 30824690 PMC6397210

[ref40] Uno S.-n.; Kamiya M.; Morozumi A.; Urano Y. A green-light-emitting, spontaneously blinking fluorophore based on intramolecular spirocyclization for dual-colour super-resolution imaging. Chem. Commun. 2018, 54 (1), 102–105. 10.1039/C7CC07783A. 29214255

[ref41] Tyson J.; Hu K.; Zheng S.; Kidd P.; Dadina N.; Chu L.; Toomre D.; Bewersdorf J.; Schepartz A. Extremely bright, near-IR emitting spontaneously blinking fluorophores enable ratiometric multicolor nanoscopy in live cells. ACS Cent. Sci. 2021, 7 (8), 1419–1426. 10.1021/acscentsci.1c00670. 34471685 PMC8393207

[ref42] Schepers A. V.; Lorenz C.; Köster S. Tuning intermediate filament mechanics by variation of pH and ion charges. Nanoscale 2020, 12 (28), 15236–15245. 10.1039/D0NR02778B. 32642745

[ref43] Endesfelder U.; Malkusch S.; Fricke F.; Heilemann M. A simple method to estimate the average localization precision of a single-molecule localization microscopy experiment. Histochem. Cell Biol. 2014, 141, 629–638. 10.1007/s00418-014-1192-3. 24522395

