# Supplementary material for: Multiplexed Nanoscopy via Buffer Exchange
Source: ACS Nano. 2024 Aug 15;18(34):23445–56. doi: 10.1021/acsnano.4c06829 (PMC11363122; doi:10.1021/acsnano.4c06829)
Supplement: Supplementary file 1 — nn4c06829_si_001.pdf [file nn4c06829_si_001.pdf]

Supporting Information

Multiplexed Nanoscopy via Buffer Exchange

Ting-Jui Ben Chang[1,2,3,4], T. Tony Yang*[1,2]

[1]Department of Electrical Engineering, National Taiwan University, Taipei, 10617, Taiwan

[2]Graduate Institute of Biomedical Electronics and Bioinformatics, National Taiwan University, Taipei, 10617, Taiwan

[3]Department of Physics, National Taiwan University, Taipei, 10617, Taiwan

[4]Nano Science and Technology Program, Taiwan International Graduate Program, Academia Sinica and National Taiwan University, Taipei, 10617, Taiwan

*Corresponding author:

T. Tony Yang

tonyyang@ntu.edu.tw

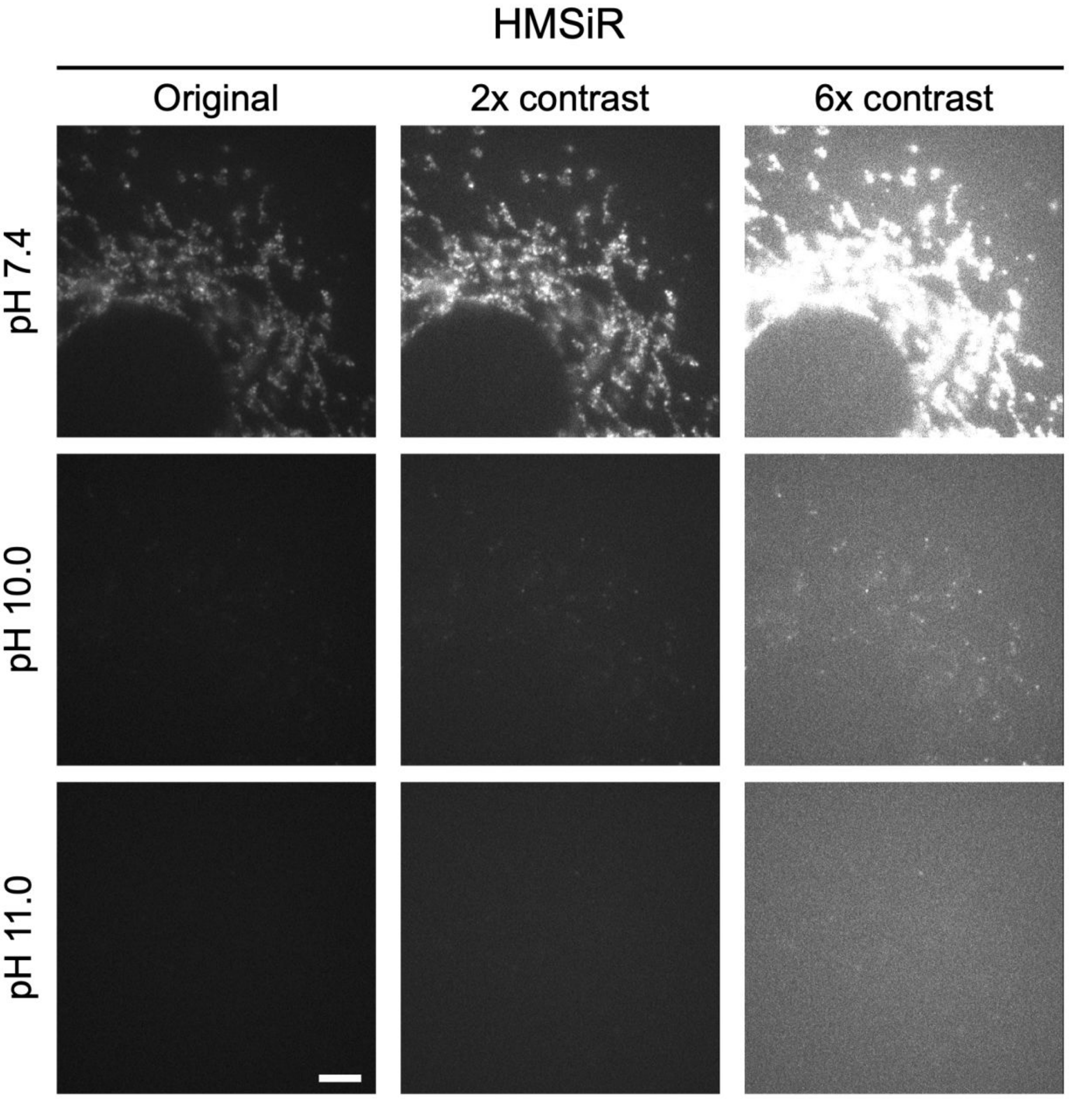

Figure S1. Effect of pH value on fluorescence intensity and photoblinking of HMSiR. Images of the mitochondrial membrane labeled with HMSiR under different pH conditions within an RPE-1 cell. The findings showed a significant decrease in both the fluorescence intensity and photoblinking of HMSiR as the pH level of the buffer solution increases. Image contrast was adjusted by factors of two and six for enhanced visual comparison. Scale bar, 5 μm.

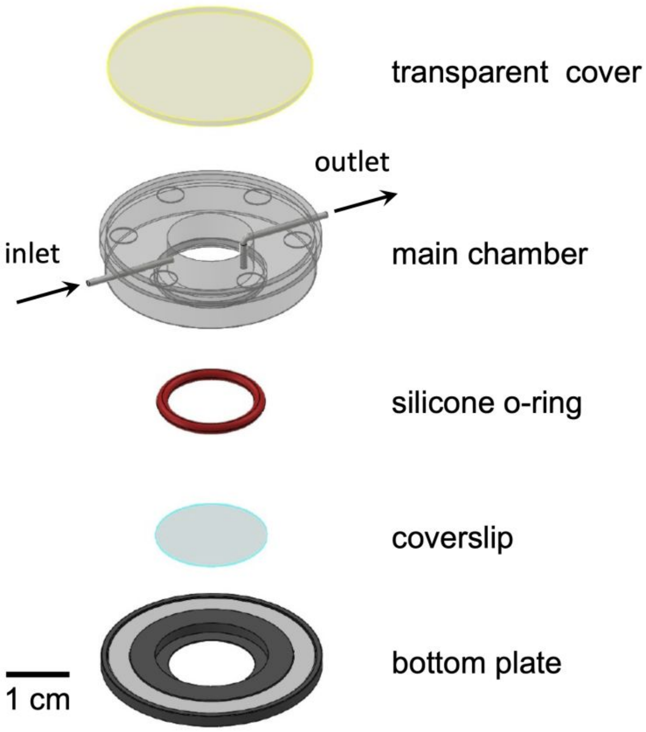

Figure S2. Sample chamber for conducting buffer exchanges. To streamline the buffer-exchange process for beSTORM, we utilized a commercially available magnetic sample chamber featuring a unidirectional inlet and outlet (CM-B18-1, Live Cell Instrument). Upon assembling all components, the system forms a closed configuration, optimizing STORM imaging in the thiol-containing buffer (TB) within an oxygen-scavenged environment. Scale bar, 1 cm.

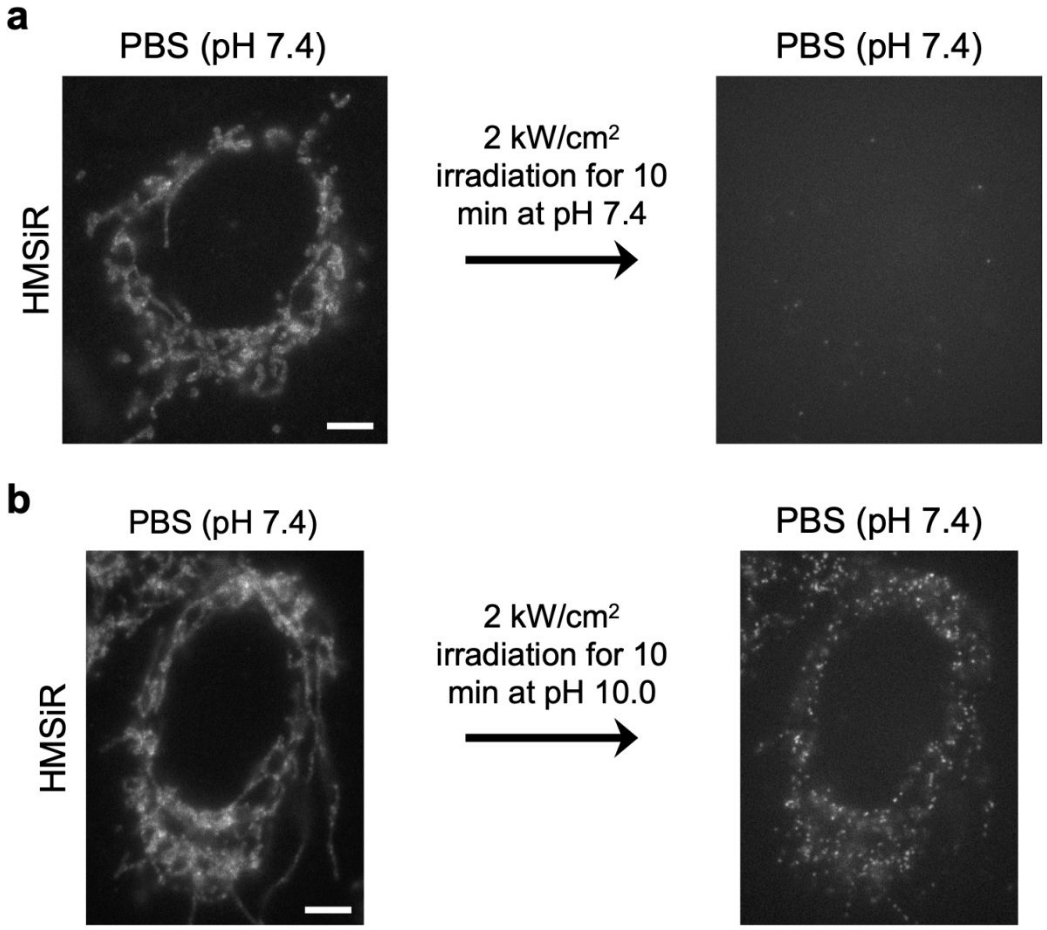

Figure S3. Protective role of high-pH buffer for HMSiR during high-power laser irradiation. **a**, **b** RPE-1 cells were immunolabeled with HMSiR for the outer mitochondrial membrane and imaged. Following initial imaging in PBS (left panel:1000 ms exposure time), cells were subjected to high-power irradiation (2 kW/cm^2) for 10 minutes under pH values of 7.4 (**a**) and 10.0 (**b**). Subsequently, cells were re-imaged for visual comparison (right panel: 20 ms exposure time). **a**, At pH 7.4, most HMSiR molecules were photobleached after prolonged high-power laser irradiation. **b**, At pH 10.0, most HMSiR molecules were retained after high-power laser irradiation. In addition to the effects of high-pH value on HMSiR demonstrated in Figure S1, this result reveals that the high-pH buffer condition provides a protective environment for HMSiR against long high-power laser irradiation (STORM imaging condition for AF647). Scale bar, 5 μm (**a**, **b**).

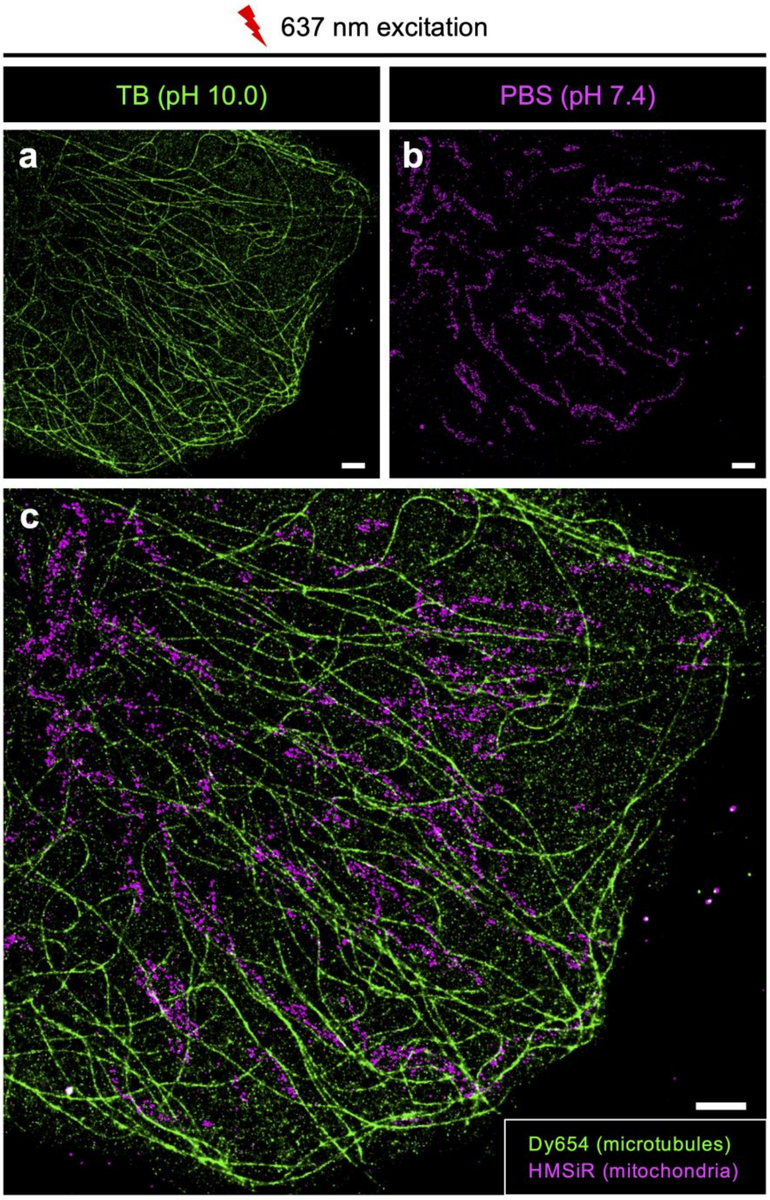

Figure S4. Far-red beSTORM imaging with Dy654 and HMSiR. **a**, **b**, Individual channels of a dual-target beSTORM result showing the superresolved microtubules (Dy654) (**a**) and mitochondrial membrane (HMSiR) (**b**) in an RPE-1 cell. **c**, Merged image from (**a** and **b**). Scale bars, 2 μm (**a**-**c**).

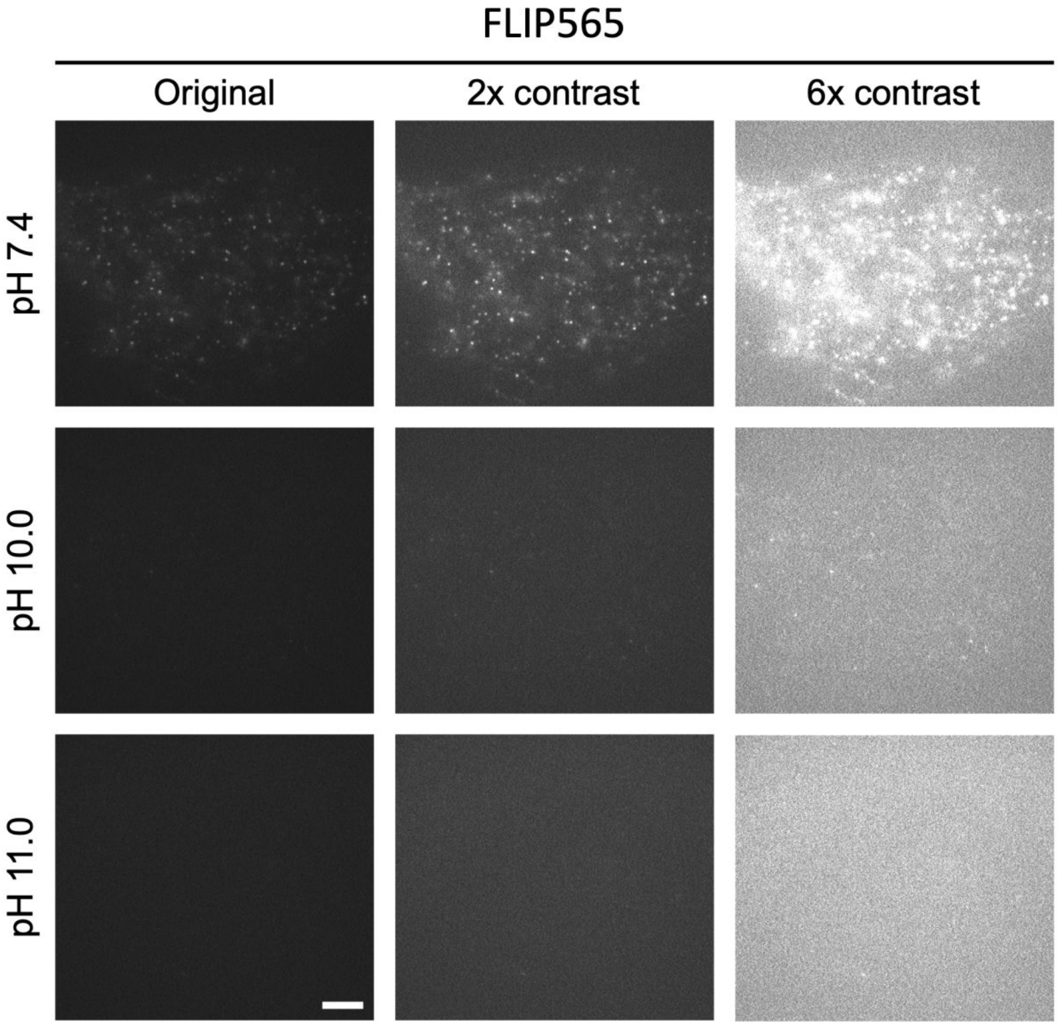

Figure S5. Effect of pH value on fluorescence intensity and photoblinking of FLIP565. Images of mitochondrial membrane labeled with FLIP565 for an RPE-1 cell under different pH levels. Likewise, the results indicated a reduction in both fluorescence intensity and photoblinking of FLIP565 with increasing pH of the buffer solution. Scale bar, 5 μm.

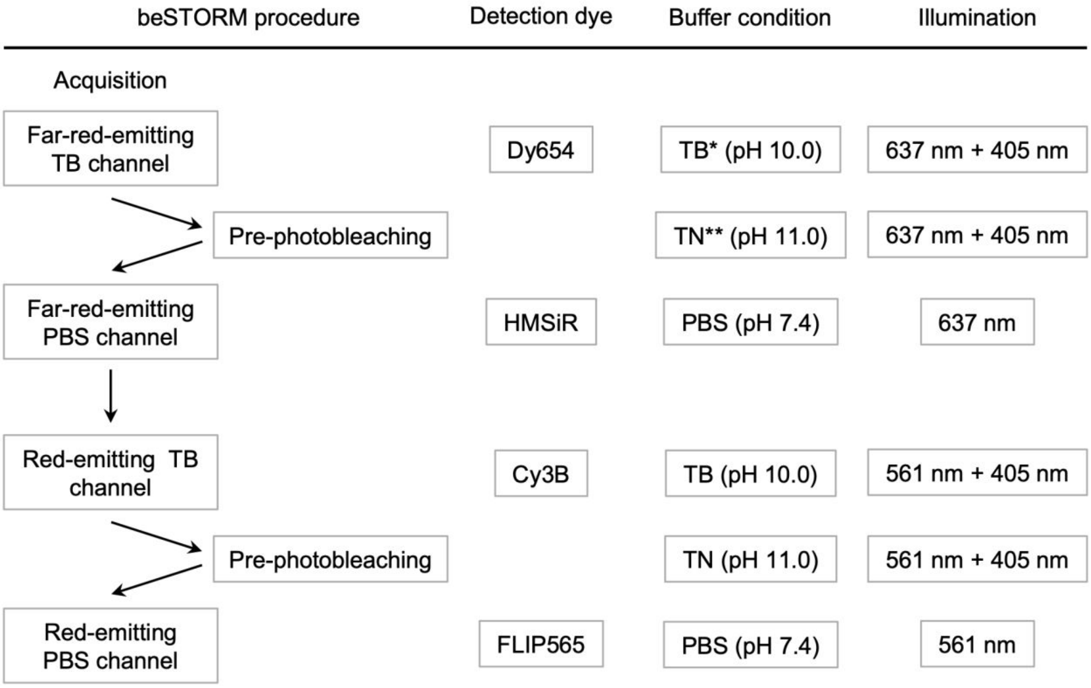

*TB: 50 mM Tris (pH 10.0), 10 mM sodium chloride (NaCl), 10 mM β-mercaptoethylamine (MEA), 10% glucose, 0.5 mg mL^{-1} glucose oxidase, and 40 μg mL^{-1} catalase

**TN: 50 mM Tris (pH 11.0) and 10 mM NaCl.

Figure S6. Four-target beSTORM procedures. beSTORM doubles the accommodated targets of SMLM in a single emitting color. With two excitation lights, beSTORM simplifies achieving four-target SMLM imaging through direct buffer exchanges. In summary, the beSTORM imaging protocol involves sequentially recording single-molecule blinking signals from far-red-emitting TB, far-red-emitting PBS, red-emitting TB, to red-emitting PBS channels, with two pre-photobleaching steps between TB and PBS channel transitions. This specific imaging sequence ensures precise identification of the detection target in its respective channel. The corresponding buffer and illumination conditions within each procedure are outlined.

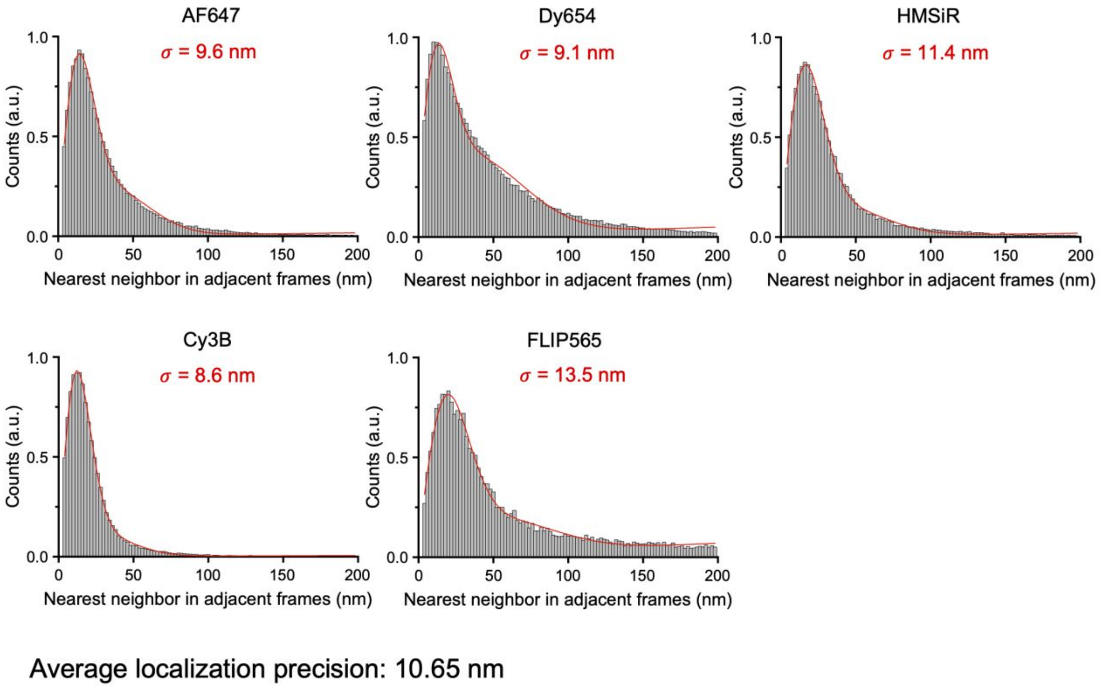

Figure S7. Localization precision assessment of beSTORM. The localization of individual single molecules was derived from the data presented in Figure S8, illustrating beSTORM imaging of the mitochondrial membrane labeled with various dyes. Localization precision was evaluated using nearest neighbor analysis (NeNA)[1] for each dye within its respective detection channel and determined by the peak of the histogram for each dye. The analysis yielded the average localization precision of 10.65 nm. Notably, AF647, Dy654, and Cy3B maintained a precision of less than 10 nm even in high-pH conditions.

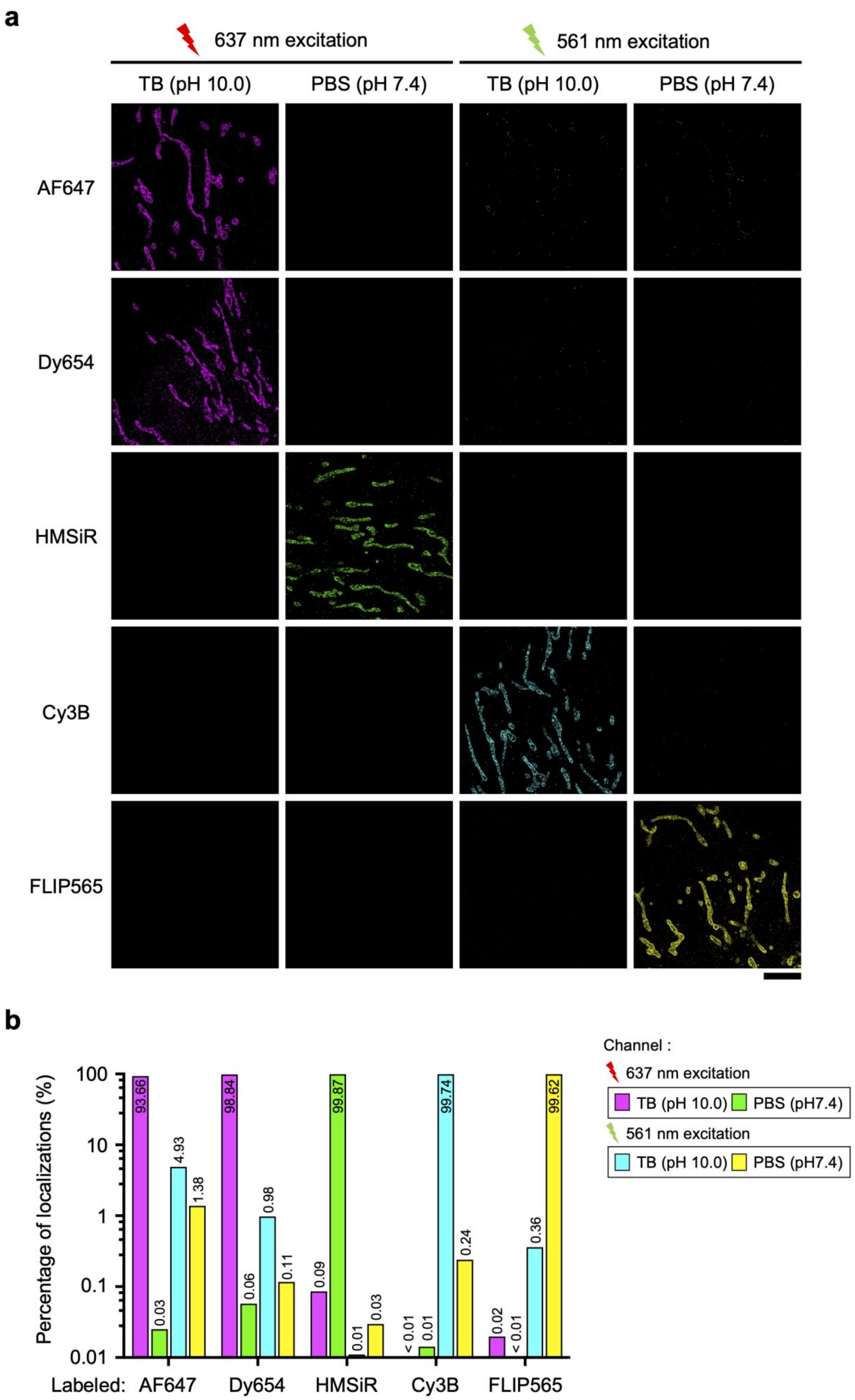

a
637 nm excitation
561 nm excitation
TB (pH 10.0)
PBS (pH 7.4)
TB (pH 10.0)
PBS (pH 7.4)
AF647
Dy654
HMSiR
Cy3B
FLIP565
b
Channel :
637 nm excitation
TB (pH 10.0)
PBS (pH7.4)
561 nm excitation
TB (pH 10.0)
PBS (pH7.4)
Percentage of localizations (%)
100
10
1
0.1
0.01
93.66
0.03
4.93
1.38
98.84
0.06
0.98
0.11
0.09
99.87
0.01
0.03
< 0.01
0.01
99.74
0.24
0.02
< 0.01
0.36
99.62
Labeled:
AF647
Dy654
HMSiR
Cy3B
FLIP565

Figure S8. Crosstalk analysis of the four-target beSTORM. To quantify the crosstalk, RPE-1 cells were immunolabeled with AF647, Dy654, HMSiR Cy3B, or FLIP565 targeting the outer mitochondrial membrane protein, then imaged separately in four channels using beSTORM. **a**, beSTORM images of the specified dyes in each channel. In addition to the channel detecting the desired proteins, signals appearing in other channels contributed to the crosstalk in beSTORM. **b**, Crosstalk among the four channels from (**a**) represented as a percentage of localizations on a logarithmic scale. The results reveal crosstalk fractions of less than 1% across all four channels when utilizing Dy654, HMSiR, Cy3B, and FLIP565 for four-target beSTORM imaging. Scale bar, 5 μm (**a**).

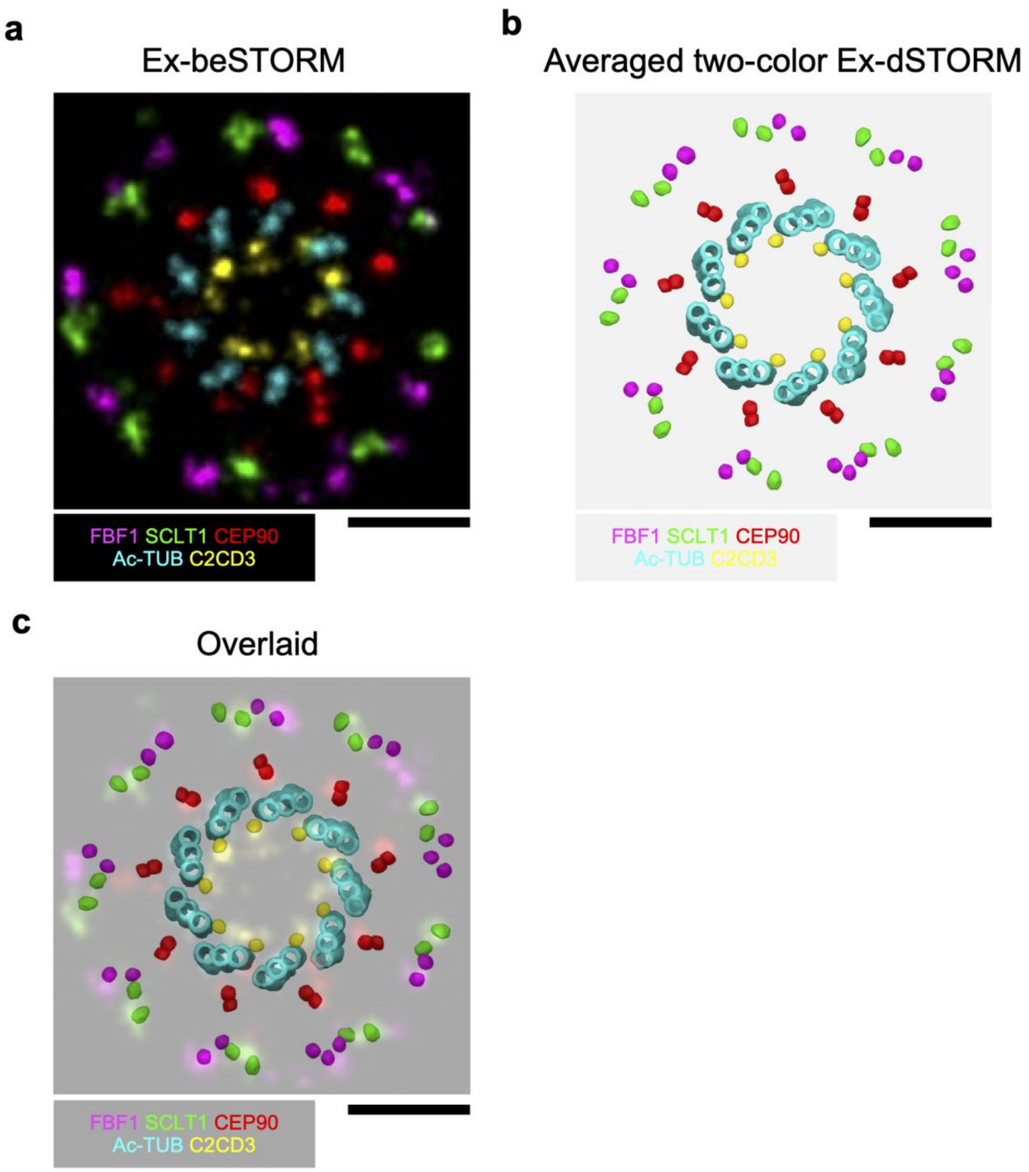

Figure S9. Comparing the five-target Ex-beSTORM image with a previously reconstructed DA model. a, A representative top-view image of five DA proteins acquired with Ex-beSTORM (repeated from Fig. 5h). **b**, Ultra-resolved model of the same proteins as depicted in (**a**), regenerated based on the averaged data in our recent study,[2] which included four experimental sets from at least three independent rounds of two-color Ex-dSTORM imaging. **c**, Overlaying images (**a**) and (**b**) by aligning them with the centriole marker (cyan). This suggests a consistent structural interpretation from both approaches, whereas Ex-beSTORM allows the acquisition of those proteins from a single round of nanoscopy imaging. Scale bars, 500 nm (**a**-**c**).

Table S1. Evaluation of red-emitting dyes on beSTORM. We compared two parameters for several synthetic dyes recognized for their excellent photoblinking properties in STORM imaging.[3-5] Cy3B is considered a potential dye candidate due to its effective quenching in PBS under high-power irradiation, while also maintaining fluorescence intensity in high-pH imaging buffers without undergoing substantial reduction.

	Quenching efficiency in PBS (pH 7.4)	Fluorescence reduction in TN buffer (pH 10.0)
Cy3B (571 nm)	Very good	Not significant
CF568 (583 nm)	Fair*	Not significant
Dy547 (565 nm)	Good	Not significant
CF583r (609 nm)	Good	Significant**

*CF568 exhibited resistance to entire photobleaching (a small amount of photoblinking was still observed after long-term high-power irradiation), which could potentially result in crosstalk.

**CF583r experienced a substantial decrease in intensity when exposed to a high-pH buffer.

Table S2. List of primary antibodies used in this study

Designation of antibody	Host species	Source	Catalog number	beSTORM dilution	Ex-beSTORM dilution
α-tubulin	Mouse IgG1	Santa Cruz	sc32293	1:250	-
α-tubulin	Rat IgG	Abcam	ab6160	1:100	-
TOMM20	Rabbit IgG	Abcam	ab186735	1:250	-
Vimentin	Chicken IgY	Abcam	ab24525	1:200	-
PMP70	Mouse IgG1	Sigma-Aldrich	SAB4200181	1:100	-
Acetyl-α-Tubulin (Ac-Tub)	Mouse IgG2b	Invitrogen	32-2700	-	1:500
C2CD3	Rabbit IgG	Sigma-Aldrich	HPA040433	-	1:200
SCLT1	Rat IgG	Tanos et al.[6]	-	1:200	1:100
FBF1	Rabbit IgG	Proteintech	11531-1-AP	-	1:150
CEP128	Rabbit IgG	Abcam	ab118797	-	1:200
ALMS1	Rabbit IgG	Bethyl	A301-815A	-	1:500
ARL13B	Rabbit IgG	Proteintech	17711-1-AP	-	1:200
ARL13B	Mouse IgG2a	Abcam	ab136648	1:500	-
TMEM67 (MKS3)	Rabbit IgG	Proteintech	13975-1-AP	1:200	-
Centriolin	Mouse IgG1	Santa Cruz	sc-365521	1:200	1:100
CEP90 (PIBF1)	Rabbit IgG	Proteintech	14413-1-AP	-	1:150
ATP synthase	Mouse IgG2b	Abcam	ab109867	-	1:150

References

(1) Endesfelder, U.; Malkusch, S.; Fricke, F.; Heilemann, M. A simple method to estimate the average localization precision of a single-molecule localization microscopy experiment. *Histochemistry and cell biology* **2014**, *141*, 629-638.

(2) Chang, T.-J. B.; Hsu, J. C.-C.; Yang, T. T. Single-molecule localization microscopy reveals the ultrastructural constitution of distal appendages in expanded mammalian centrioles. *Nature Communications* **2023**, *14* (1), 1688.

(3) Dempsey, G. T.; Vaughan, J. C.; Chen, K. H.; Bates, M.; Zhuang, X. Evaluation of fluorophores for optimal performance in localization-based super-resolution imaging. *Nature methods* **2011**, *8* (12), 1027-1036.

(4) Wang, B.; Xiong, M.; Susanto, J.; Li, X.; Leung, W. Y.; Xu, K. Transforming Rhodamine Dyes for (d) STORM Super-Resolution Microscopy via 1, 3-Disubstituted Imidazolium Substitution. *Angewandte Chemie International Edition* **2022**, *61* (9), e202113612.

(5) Lehmann, M.; Lichtner, G.; Klenz, H.; Schmoranzer, J. Novel organic dyes for multicolor localization-based super-resolution microscopy. *Journal of biophotonics* **2016**, *9* (1-2), 161-170.

(6) Tanos, B. E.; Yang, H.-J.; Soni, R.; Wang, W.-J.; Macaluso, F. P.; Asara, J. M.; Tsou, M.-F. B. Centriole distal appendages promote membrane docking, leading to cilia initiation. *Genes & development* **2013**, *27* (2), 163-168.
